# Genomic evolution and virulence association of *Clostridioides difficile* sequence type 37 (ribotype 017) in China

**DOI:** 10.1080/22221751.2021.1943538

**Published:** 2021-06-14

**Authors:** Xingxing Xu, Yuo Luo, Huan Chen, Xiaojun Song, Qiao Bian, Xianjun Wang, Qian Liang, Jianhong Zhao, Chunhui Li, Guangzhong Song, Jun Yang, Lingli Sun, Jianmin Jiang, Huanying Wang, Bo Zhu, Guangyong Ye, Liang Chen, Yi-Wei Tang, Dazhi Jin

**Affiliations:** a Department of Clinical Laboratory, Women’s Hospital, Zhejiang University School of Medicine, Hangzhou, People’s Republic of China; b School of Laboratory Medicine, Hangzhou Medical College, Hangzhou, People’s Republic of China; c School of Biotechnology and Biomolecular Sciences, University of New South Wales, Sydney, Australia; d Key Laboratory of Microorganism technology and bioinformatics research of Zhejiang Province, Hangzhou, People’s Republic of China; e NMPA Key Laboratory For Testing and Risk Warning of Pharmaceutical Microbiology, Hangzhou, People’s Republic of China; f Centre of Laboratory Medicine, Zhejiang Provincial People’s Hospital, People’s Hospital of Hangzhou Medical College, Hangzhou, People’s Republic of China; g Zhejiang Provincial Center for Disease Control and Prevention, Hangzhou, People’s Republic of China; h Department of Laboratory Medicine, Hangzhou First People’s Hospital, Hangzhou, People’s Republic of China; i Department of Clinical Microbiology, Second Hospital of Hebei Medical University, Hebei Provincial Center for Clinical Laboratories, Shijiazhuang, People’s Republic of China; j Infection Control Center, Xiangya Hospital of Central South University, Changsha, People’s Republic of China; k Center for Discovery and Innovation, Hackensack Meridian Health, Nutley, NJ, USA; l Department of Medical Sciences, Hackensack Meridian School of Medicine, Nutley, NJ, USA; m Cepheid, Danaher Diagnostic Platform, Shanghai, People’s Republic of China

**Keywords:** *Clostridioides difficile*, ST37, whole genome sequencing, phylogeny, severe CDI

## Abstract

*Clostridioides difficile* sequence type (ST) 37 (ribotype 017) is one of the most prevalent genotypes circulating in China. However, its genomic evolution and virulence determinants were rarely explored. Whole-genome sequencing, phylogeographic and phylogenetic analyses were conducted for *C. difficile* ST37 isolates. The 325 ST37 genomes from six continents, including North America (*n* = 66), South America (*n* = 4), Oceania (*n* = 7), Africa (*n* = 9), Europe (*n* = 138) and Asia (*n* = 101), were clustered into six major lineages, with region-dependent distributions, harbouring an array of antibiotic-resistance genes. The ST37 strains from China were divided into four distinct sublineages, showing five importation times and international sources. Isolates associated with severe infections exhibited significantly higher toxin productions, *tcdB* mRNA levels, and sporulation capacities (*P* < 0.001). Kyoto Encyclopedia of Genes and Genomes analysis showed 10 metabolic pathways were significantly enriched in the mutations among isolates associated with severe CDI (*P* < 0.05). Gene mutations in glycometabolism, amino acid metabolism and biosynthesis virtually causing instability in protein activity were correlated positively to the transcription of *tcdR* and negatively to the expression of toxin repressor genes, *ccpA* and *cod*Y. In summary, our study firstly presented genomic insights into genetic characteristics and virulence association of *C. difficile* ST37 in China. Gene mutations in certain important metabolic pathways are associated with severe symptoms and correlated with higher virulence in *C. difficile* ST37 isolates.

## Introduction

Globally, *Clostridioides difficile* has emerged as a leading cause of antibiotic-associated diarrhoea, attributed to the production of two potent toxins, TcdA and TcdB [1]. Initially, it was thought that all toxigenic *C. difficile* strains expressed two major toxins, TcdA and TcdB, because the significance of TcdA-negative and TcdB-positive strains was not obvious. However, it was later found that A^-^B^+^ strains also induced pseudomembranous colitis as A^+^B^+^ strains [2], and TcdB, which is more potent than TcdA in damaging human colonic epithelium, could conduct its cytotoxic effect in the absence of TcdA [3]. As one of A^-^B^+^ strains, *C. difficile* sequence type (ST) 37, corresponding to ribotype 017 (RT017), lacks *tcdA* expression, but this strain results in widespread clinical *C. difficile* infections (CDI) [4–7], and furthermore ST37 (RT017) causes clinical symptoms as severe as that caused by “hypervirulent” ST1(RT027) [8]. It has been reported that this genotype was associated with lethal CDIs in Germany [9] and severe CDIs in China [10]. Thus, A^-^B^+^ *C. difficile* ST37(RT017) has been recognized as the important genotype with the clinical significance recently.

Over the last two decades, *C. difficile* ST37(RT017) has been isolated from many regions in the world [11]. In the late 1990s, ST37(RT017) infection was reported with CDI outbreaks in several countries, and the first group of CDI outbreaks was reported from 1995 to 1998 in Canada, Netherlands, and Poland [11]. Of them, clinical features of the patients were only described in Canada with a 31.3% of recurrent cases and a 37.5% mortality rate [2,12]. Moreover, lots of non-outbreak events associated with ST37(RT017) occurred worldwide, especially in Asia with high prevalence [11]. ST37(RT017) is obviously endemic in Asia and has existed in this region for a long time, and this genotype is also reported to be one of the predominant toxigenic strains in lots of countries in Asia [11]. Based on the recent reports [10,13], ST37 genotype has been prevalent in China, and these strains show greater resistance to multiple antibiotics than those with other STs, leading to complicated CDI cases with severe symptoms being reported in China.

Whole genome sequencing (WGS) has been used to perform phylogenetic analysis to investigate genetic relationships among *C. difficile* isolates derived from different sources, and to explore strain transmission and genome evolution [14–17]. So far, the genomes of two *C. difficile* ST37(RT017) strains (CF5 isolated in Belgium and M68 isolated in Ireland) have been completely sequenced as the important reference genomes for WGS studies [11]. Previous phylogenetic analysis indicated that globally two evenly split *C. difficile* ST37 sublineages of animal and human origin were differentiated by four single-nucleotide polymorphisms (SNPs) [18]. However, molecular evolution and transmission of *C. difficile* ST37 strains in the ST37 epidemic region, i.e. China, are unclear. Recent data show that *C. difficile* ST37 is a predominant genotype, and frequently leads to the presentation of severe clinical symptoms in China [10,13,19], whereas the genomic characteristics underlying the clinical progression within ST37 *C. difficile* strains are yet to be determined.

In this study, 48 *C. difficile* ST37 isolates from North America, mainland China, and other Asia-Pacific regions were subjected to WGS. Phylogenetic analysis and virulence-associated phenotype determination were performed to investigate the genome evolution and transmission of *C. difficile* ST37 in mainland China; to study the association between the virulence and genomic characteristics of ST37 strains; and to explore the relationship of regulatory pathways of key genomic signatures with severe clinical symptoms of CDI.

## Materials and methods

### C. difficile isolates and genome sequences

A total of 48 *C. difficile* ST37 isolates were obtained between 2011 and 2017 from patients with CDIs and asymptomatic individuals from four different geographical areas, including Zhejiang (*n* = 14), Hebei (*n* = 15), Hunan (*n* = 6), and Hong Kong (*n* = 2) in China, Pusan in South Korea (*n* = 3), Singapore (*n* = 1), and New York City in the United States (*n* = 7). All isolates were sent to our laboratory in a transport culture medium and recovered on cefoxitin–cycloserine fructose agar plates (Oxoid, Basingstoke, Great Britain) by incubation for 48 h at 37°C in an anaerobic chamber with GENbag Anaer (bioMérieux, Marcy l’Étoile, France), as reported previously [20]. Raw reads data of 277 ST37 genomes from six continents, including North America (*n* = 59), South America (*n* = 4), Oceania (*n* = 7), Africa (*n* = 9), Europe (*n* = 137) and Asia (*n* = 61), with isolation dated between 1990 and 2013 [18] were downloaded from the NCBI database (up to May 2017; Bioproject numbers ERP009770 and PRJEB11868).

### Classification of CDI severities

A blinded medical chart review was conducted by two physicians, as reported previously [10,21–23]. Diarrhoea caused by other bacteria or viruses of the hosts were ruled out. CDI severities were categorized into asymptomatic carriers, mild-to-moderate CDI, and severe CDI, based on the clinical guidelines of the Infectious Disease Society of America (IDSA)/Society of Hospital Epidemiologists of America (SHEA) and European Society of Clinical Microbiology and Infectious Diseases (ESCMID) [23,24]. Briefly, asymptomatic carriers had no diarrhoea, ileus, or colitis, but carried *C. difficile*, patients with mild or moderate had leukocytosis with a white blood cell count of 15,000 cells/mL or lower and a serum creatinine level less than 1.5 times the premorbid level, severe or life-threatening CDI is defined as an episode of CDI with (one or more specific signs and symptoms of) severe colitis or a complicated course of disease, with significant systemic toxin effects and shock. An ethical approval for clinical data collection was received from the Institutional Review Boards for each respective site. This study was approved by the Ethics Committee of the Hangzhou Medical College (the ethics approved number: T-043-R), and the informed consent requirement was waived due to no more than minimal risk involved in this study.

### WGS and data assembly

Genomic DNA was extracted as previously described [25]. WGS libraries were created using the TruePrep™ DNA library prep kit V2 (Illumina, San Diego, CA, USA). WGS was performed using the Illumina Hiseq X Ten platform with 150-base paired-end reads. The sequence data were processed and quality controlled according to a standard pipeline as previously described [26]. Briefly, FASTQ-formatted sequencing reads were quality controlled with a minimum quality Phred score of 30 (as a rolling average over 4 bases) [27]. Trimmomatic version 0.36 [27] was used to remove adapters and low-quality sequences with default parameters, except MINLEN was set as 75, and 55.6 Gb of clean bases was finally generated (1.183 Gb/per isolate, Q20 ≥ 95%). The Illumina sequence reads were mapped to the *C. difficile* M68 (RT017) reference genome (GenBank: FN668375) using Bowtie2 (version 2.2.9). The genome data for the 48 isolates from this study, and the downloaded data for 277 genomes, were *de novo* assembled using Velvet (version 1.2.10). The genomic sequence data from this study were deposited in the NCBI database under study accession number PRJNA591265.

### SNP identification and homologous modelling

SAMtools (version 1.3.1) was used to identify SNPs as previously reported [28]. After removing low-confidence alleles with a consensus base quality < 20 and a read depth < 5 or a heterozygous base call, high-quality SNPs and indels were annotated using SnpEff (version 4.3). The distribution of SNPs and indels in phylogenetic lineages, CDI severities, and origins of the *C. difficile* isolates were extracted. After confirmation by Sanger sequencing, the genes with SNPs and indels that were associated with severe CDIs were analysed by Kyoto Encyclopedia of Genes and Genomes (KEGG) enrichment using KOBAS 3.0 (http://www.genome.jp/kegg/) [29,30], and translated into amino acid sequences for homologous modelling on the website (https://www.rcsb.org).

### Identification of gene recombination and phylogenetic analysis

Gene recombination was identified using Gubbins [31]. The *r/m* ratio was calculated within the deep-branching phylogeny, providing a relative probability of a nucleotide change due to recombination rather than to a point mutation. Eleven completely assembled and fully annotated *C. difficile* genomes (Table S1) were used to determine core genes. A total of 2,227 genes were defined as the core gene sets using BLASTp with an *E*-value of 1e^−10^, and orthologous proteins were then grouped using the orthoMCL algorithm. The maximum-likelihood tree topology and branch lengths were inferred using RAxML with 1,000 bootstrap replicates [32].

Bayesian evolutionary analysis was performed using BEAST [33,34] (version 2.4.7) with three clock models (strict clock and uncorrelated relaxed clocks with lognormal and exponential distributions), two population models (constant and skyline), and two site models (generalized time-reversible and Hasegawa–Kishino–Yano models). Each combined model was run three times, and good convergence of chains and effective sample size values were examined using Tracer (version 1.5). A marginal likelihood estimation was then performed for each run using path sampling and steppingstone sampling to evaluate the best-fitting model for the data by calculating the Bayes factor. For the data set used, the generalized time-reversible, strict-clock, and skyline models were optimized as the best fit for further Bayesian evolutionary analysis using BEAST, which was set to estimate the time to the most recent common ancestor of taxon groupings. BEAST was run for 100 million generations, with sampling performed every 10,000 states, to obtain the substitution rates, importation times, and divergence dates. TempEst (version 1.5.3) and the LSD version 0.3 beta programs were also used to confirm the molecular clock generated by BEAST. Moreover, phylogeographic spreading events were inferred by BEAST, as described above.

### Transposable elements and antibiotic resistance-related genes

The transposable genetic elements (http://transposon.lstmed.ac.uk/tn-registry) reported in previous studies [14,35–37] were mapped to genomic sequences using MUMmer (version 3.23). Antibiotic resistance-associated genes were determined using RGI (version 5.1.0) analysis and the CARD antibiotic resistance gene database (https://card.mcmaster.ca/home). Each type of *tetM* allele found in this study was designated a number through alignment of *tetM* gene nucleotide sequences available at https://pubmlst.org/bigsdb?db=pubmlst_cdifficile_seqdef&page=downloadAlleles [16].

### Antibiotic susceptibility testing

The minimal inhibitory concentrations **(**MICs) were determined for 12 antibiotics, namely, vancomycin, metronidazole, moxifloxacin, erythromycin, clindamycin, rifampicin, levofloxacin, gatifloxacin, ciprofloxacin, fusidic acid, tetracycline, and piperacillin/tazobactam, using an agar dilution method according to the M11-A8 standard [38]. Reference strains of *Bacteroides fragilis* (ATCC 25285) and C. difficile (ATCC 700057) (nontoxigenic [*tcdA*- and *tcdB*-], ribotype 038) were included in each test as quality controls. The MIC breakpoints were interpreted as reported previously [10,38]. A strain with resistance to at least three classes of antibiotics was defined as multidrug resistant, as previously described [39].

### Measurement of C. difficile toxin concentration and sporulation ability

The total toxin concentration was quantified using the fluorescence-based VIDAS *C. difficile* toxin A & B enzyme immunoassay (bioMérieux), as reported previously [40]. Briefly, the 48 isolates were inoculated in brain heart infusion broth for 24 h at 37 °C in an anaerobic chamber with GENbag Anaer (bioMérieux). The culture samples were adjusted to OD600 = 1.0 and centrifuged. The supernatant (300 μL) was transferred to the sample well and measured according to the manufacturer’s instruction. The concentration of the total toxins was proportioned to the final ﬂuorescence intensity. The sporulation capacity was measured as reported previously [40]. Briefly, the *C. difficile* culture samples were incubated for 24 h and adjusted to approximately OD600 = 1.0. One-millilitre of the isolates was heated at 60°C for 25 min, and serial dilutions were inoculated onto 2% brain heart infusion agar plates with 0.1% taurocholate [40]. After 24 h of anaerobic incubation, the heat-resistant cells were counted in a range between 30 and 300 spores per plate. All the experiments were repeated three times.

### Relative quantification of mRNA expression of toxin-related genes

The 48 *C. difficile* isolates were incubated in brain heart infusion broth at 37°C in an anaerobic chamber with GENbag Anaer (bioMérieux). Total RNA was extracted at early stationary phase (after approximately 11 h of growth). Bacterial RNA was extracted using the RNeasy mini kit (Qiagen, Hilden, Germany) according to the manufacturer’s instructions. The primer sets for five genes (*tcdB*, *tcdR*, *codY*, *ccpA*, and 16S rRNA) are shown in Table S2. Reverse transcription and real-time quantitative PCR were performed as described previously [41]. The 16S rRNA gene was used as an endogenous control to normalize the expression levels of the other genes, and the results were calculated using the comparative cycle threshold method [41]. The reference *C. difficile* strains (ATCC BAA-1870, ST1, ribotype 027) and (ATCC700057, ribotyping 038) were used as a positive control and negative control, respectively, for the expression of the *tcdB* and toxin-related genes. All the experiments were repeated three times, each experiment run independently and different colonies from one isolate were tested.

### Data analysis

Data were analysed using the SPSS version 22.0 software (SPSS, Inc., Chicago, IL, USA). Clinical information of the 48 patients with severe to mild CDI and the indel frequencies and SNP mutation rate of China and other countries were analysed using the χ^2^ test. Differences in toxin concentrations and sporulation capacities among the three CDI severity groups and the relative mRNA expression levels of severe CDI with mutations, non-severe CDI and severe CDI without mutations groups were analysed using the nonparametric Kruskal–Wallis *H* test; the Mann–Whitney *U*-test or a *t*-test were further used for pairwise group comparison. The Bonferroni test was used to adjust the significance level. The pathway enrichment of corrected *P* value was analysed using the Benjamini-Hochberg method [42]. Relative transcription levels were calculated using the 2^−ΔΔCt^ method [43]. *P*-values < 0.05 were considered statistically significant.

## Results

### Genomic characteristics of C. difficile ST37

All the 325 WGS data were analysed after sequence quality control and mapping to the *C. difficile* strain M68. Firstly, truncate *tcdA* and intact *tcdB* were identified among all ST37 strains as previously reported [14], and other virulence genes were randomly scattered in ST genomes (data not shown). Then, 13,195 high-quality SNPs were identified after the resulting alignments. Gubbins analysis revealed 11,473 spatially clustered SNPs within 186 homologous recombination events. After removing these SNPs, 1,722 high-quality biallelic SNPs were extracted. Of these, 703 non-rare (core) SNPs (40.8%, 703/1,722) were present in the core genome, which included 546 (77.7%, 546/703) non-synonymous SNPs. A total of 127 (18.1%, 127/703) non-rare SNPs were found to be specific to the ST37 isolates from China, of which 76.4% (97/127) were non-synonymous mutations. The mutation rates of the Chinese and all other countries ST37 isolates were estimated to be 3.56–9.73 × 10^−4^ and 4.38–6.12 × 10^−4^ per site per year, respectively. Fifty-two recombination events were also found in 325 ST37 isolates (data not shown). The recombination-to-mutation (*r/m*) ratios for all *C. difficile* ST37 and Chinese ST37 isolates were approximately 6.66 and 7.49, respectively. More than half of the ST37 genomes (54.8%, 178/325) had indel frequencies of 10–15%, while 40.3% (131/325) of isolates demonstrated indel frequencies of 5–10%. Among them, 4.6% (15/325) of the genomes had indel frequencies over 15%, and they were all from Asia, including 11 Chinese isolates. Only one isolate (0.3%, 1/325) with an indel frequency between 1 and 5% was from China, while 98.4% (62/63) of the Chinese isolates had indel frequencies of more than 10% (Figure S1). Our results suggested that ST37 strains from China had relative higher SNP mutation and indel frequencies in comparison to their global ST37 counterparts. The ST37 strains with indel frequencies ≥10% in China was significantly more than other countries (*χ*^2 ^= 19.84, *P* < 0.001). The ST37 strains with the SNP mutation rate of 1- 3% in China was significantly more than other countries (*χ*^2^ = 14.70, *P *= 0.001).

Thirteen transposons, namely, Tn*916*, Tn*1116* (from the Tn*916* family), Tn*6194* (from a Tn*916*-like family), Tn*6218* and Tn*5397* (closely related to Tn*916*), Tn*2010*, Tn*2009*, and Tn*6002* (Tn*916*-like elements), Tn*5251* (from the Tn*916*/Tn*1545* family of conjugative transposons), Tn*6247* and Tn*6248* (Tn*5397*-like elements), Tn*6003* (Tn*6002*-like element), and Tn*4453*, were found to be widely dispersed in the ST37 genomes (Figure S2). The estimated insertion times for four transposons (Tn*916*, Tn*5251*, Tn*6247*, and Tn*6248*) were in ∼1977, while the insertion time for Tn*6218* was approximately after 2005. No exact insertion times were detected for the remaining transposons dispersed in ST37 genomes.

**Figure 1.**
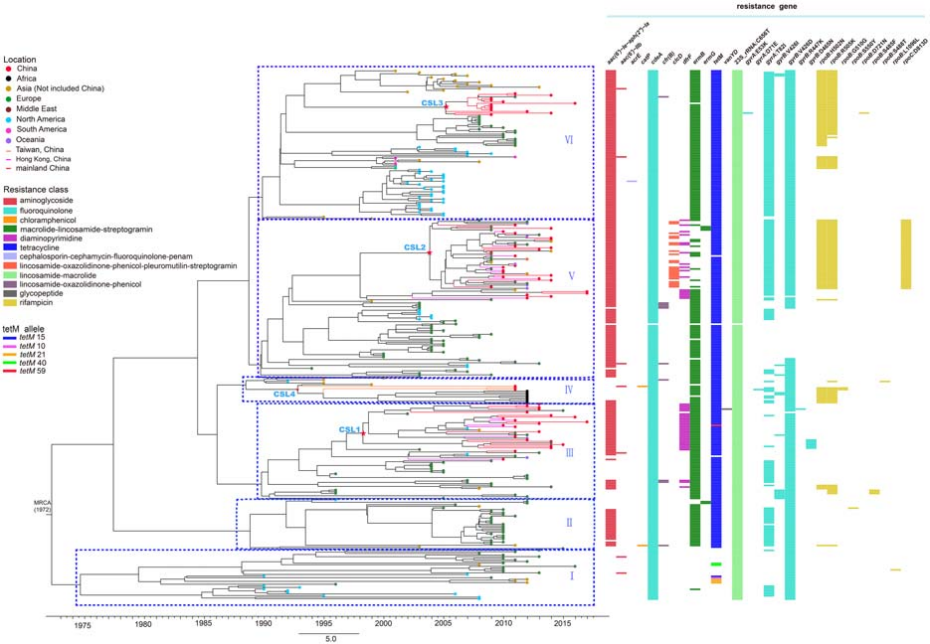
The evolution of the global 325 ST37 genomes through time was oriented by using a geotemporal model. Six major lineages were defined as lineages I–VI. *C. difficile* ST37 strains from China were clustered into four sublineages (CSL). Terminal nodes were coloured according to different countries or regions. The X axis was the isolation year. Coloured bars to the right of the phylogeny indicate the presence of 12 putative antibiotic resistance genes and 17 antibiotic resistance associated genes with different amino acid substitutions. The presence of the five *tetM* alleles (*tetM* 10, 15, 21, 40 and 59) was indicated by the coloured bars to the column of *tetM*.

A total of 29 putative antibiotic-resistance genes, including 17 resistance gene variants with amino acid substitutions, were identified within the 325 genomes (Figure 1). The gene for tetracycline resistance, *tetM*, located on the transposons of Tn*916*, Tn*6247*, Tn*6248*, Tn*5251*, Tn*1116*, Tn*5397,* and Tn*2009*, was present in 91.7% (298/325) of *C. difficile* isolates. Among the *tetM*-positive isolate genomes, *tetM* 15 was the most predominate allele (97.7%, 291/298), followed by *tetM* 21 (1.0%, 3/298), *tetM* 40 (0.7%, 2/298), *tetM* 10 (0.3%, 1/298), and *tetM* 59 (0.3%, 1/298) (Figure 1). For macrolide–lincosamide–streptogramin B (MLS_B_) resistance, the *ermB* gene was identified in Tn*6002*, Tn*6003*, Tn*2010*, Tn*6218*, Tn*1116,* and Tn*6194*, and scattered in 77.8% (253/325) of strains, but a mutation (C656T) in 23S rRNA gene was detected in all 325 strains. For fluoroquinolone resistance, the *cdeA* gene was detected in all 325 strains, and the common substitutions were found in *gyrA* (T82I) observed in 62.8% (204/325) and *gyrB* (V426D) in 90.8% (295/325) of the isolates. Two main substitutions in *rpoB* associated with rifampicin resistance were found with 34.8% (113/325) of H502N and 34.2% (111/325) of R505K. One of the chloramphenicol resistance genes, *cfr(B)* in Tn*6218*, was found in ten strains including eight from Europe and two from Asia. Chromosome-encoded dihydrofolate reductase trimethoprim resistance gene, *dfrF*, was found in the 48 isolates, mainly from China (43.8%, 21/48) and Europe (35.4%, 17/48). The *aac(6′)-ie-aph(2″)-ia* cassette associated with aminoglycoside resistance was found in 76.6% (249/325) of strains, respectively. The above results presented a wide distribution of antibiotic-resistance genes in *C. difficile* ST37 strains.

### Molecular evolution of global ST37 isolates

Bayesian evolutionary analysis by sampling trees (BEAST) indicated that the 325 global ST37 genomes were divided into six major lineages (I–VI) (Figure 1), primarily differentiated by 18 unique SNPs (Table S3). The distribution of strains in each of the six lineages was noticeably region dependent. Most of the ST37 strains from North America were in lineages I and VI. All strains from Africa and Taiwan were aggregated in lineage IV. Most strains in lineage II were from Europe, and strains in lineages III and V mainly originated in Europe and Asia, respectively. The origins of strains in lineage VI were mainly Asia, North America, and Europe, with each group located within its own lineage. BEAST allowed tracing global ST37 isolates back to ∼1972 (95% highest probability density interval 1962-1984), suggesting *C. difficile* ST37 might have developed before 1970.

**Figure 2.**
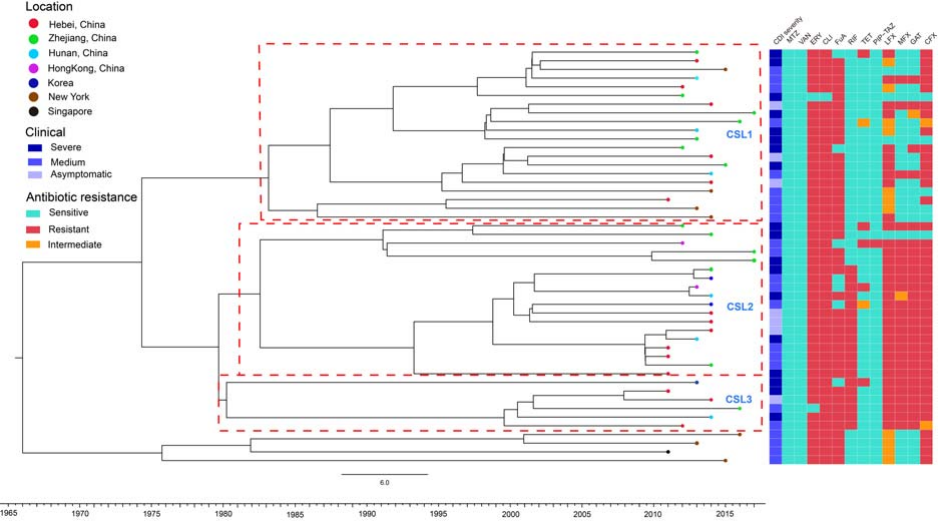
Bayesian evolutionary analysis of 48 sequenced ST37 isolates showed the three major sublineages corresponding to CSL1, CSL2 and CSL3, respectively. Terminal nodes were coloured according to different countries or regions. The X axis was the isolation year. A total of 12 antibiotic resistance phenotype were listed on the right (red: resistance; green: susceptible; orange: intermediate). metronidazole (MTZ), vancomycin (VAN), clindamycin (CLI), erythromycin (ERY), fusidic acid (FuA), rifampicin (RIF), levofloxacin (LFX), moxifloxacin (MFX), gatifloxacin (GAT), tetracycline (TET), piperacillin/tazobactam (PIP-TAZ), and ciprofloxacin (CFX). The colours from light to dark were on behalf of CDI severity shown in the middle part.

The strains in China (*n* = 63) clustered into four sublineages (Chinese sublineages 1–4, CSL1–4) based on the 14 specific SNPs (Table S4), which were mainly distributed in above-described global lineages III, V, VI, and IV, respectively (Figure 1). Four strains from Taiwan belonged to CSL4. All CSLs were widespread geographically, with no region-dependent distribution in mainland China. The 48 isolates sequenced in this study were also analysed using BEAST; the results showed the same pattern as that of the three CSLs from mainland China (Figure 2).

### Transmission of C. difficile ST37 within China

**Figure 3.**
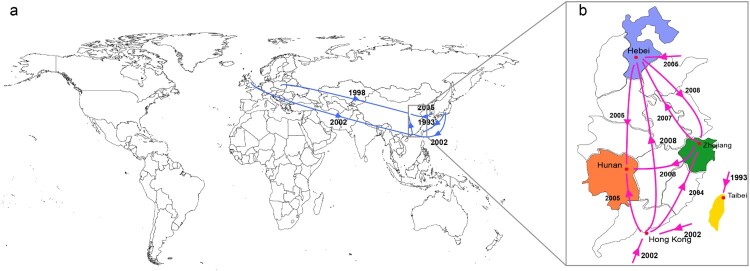
Transmission events inferred for Chinese ST37 by Bayesian evolutionary analysis. (a) Global spread (blue arrows) of Chinese ST37 inferred by phylogeographic analysis. (b) Pink arrows showed transmission routes of ST37 isolates into and within China based on phylogeographic analysis.

Geotemporal analysis of 63 strains from China revealed that the initial introduction of ST37 into China occurred via at least five independent importation events (Figure 1). The first introduction was from Japan to Taiwan in ∼1993 (lineage IV), whereas the second importation was from Europe to China (locations unclear) in ∼1998 (lineage III). The third and fourth importation events were from Japan and Europe to Hong Kong in ∼2002, respectively (lineage V), and the last event was from South Korea to Hebei province in China in ∼2005 (lineage VI) (Figure 3(a)). However, the analyses suggested that ST37 strains from Zhejiang and Hunan were not directly transmitted from outside mainland China, but likely obtained through domestic transmission. Bayesian evolutionary analysis demonstrated that long-distance geographic transmissions occurred frequently and extensively in the cases of ST37 strains from mainland China. CSL1 and CSL3 developed and spread rapidly when the ST37 genotype was introduced into mainland China. However, CSL2 emerged and gradually advanced in mainland China from 2003, one year after the introduction. Frequent ST37 strain transmissions occurred between mainland China provinces between 2005 and 2008 (Figure 3(b)).

### Antibiotic susceptibility of C. difficile ST37 strains

Antibiotic susceptibility testing was performed in the 48 isolates sequenced in this study. The detailed MICs were shown in Table 1. The rate of resistance to MLS_B_ was 97.9% (47/48), and 93.8% (45/48) of the isolates were resistant to fluoroquinolone. The rates of resistance to the fourth-generation fluoroquinolones gatifloxacin (58.3%, 28/48) and moxifloxacin (54.2%, 26/48) were lower than those to the third-generation fluoroquinolones levofloxacin (91.7%, 44/48) and ciprofloxacin (87.5%, 42/48). No isolates were resistant to vancomycin or metronidazole. The rates of resistance to MLS_B_, fluoroquinolone, and fusidic acid (85.4%, 41/48) were distinctly higher than those to rifampicin (39.6%, 19/48), tetracycline (14.6%, 7/48), and piperacillin/tazobactam (2.0%, 1/48). Besides, 91.7% (44/48) of the isolates were multidrug-resistant.

**Table 1.** The MICs for 12 antibiotic agents against the 48 isolates involved in this study.

Strain	Antibiotics^a^											
	MTZ	VAN	CLI	ERY	FuA	RIF	LFX	MFX	GAT	TET	PIP-TAZ	CFX
China-HB1	0.125	0.125	>256	>256	2	>128	>128	16	16	4	8	16
China-HB2	0.25	0.125	>256	>256	2	≤0.06	4	1	0.5	4	4	16
China-HB3	0.06	0.06	>256	64	2	>128	>128	16	16	2	1	8
China-HB4	0.03	0.125	>256	>256	1	>128	>128	16	32	4	8	16
China-HB5	0.03	0.06	>256	64	2	64	8	16	8	0.25	8	8
China-HB6	0.25	0.25	128	128	1	>128	8	16	32	0.125	1	4
China-HB7	0.25	0.5	>256	>256	1	2	4	1	0.5	2	1	32
China-HB8	0.125	0.5	256	256	1	1	4	1	0.25	0.25	1	16
China-HB9	0.125	0.5	256	128	1	1	>128	16	32	0.125	1	16
China-HB10	0.25	0.5	256	>128	1	>128	>128	8	16	1	8	16
China-HB11	0.125	0.25	>256	>128	2	>128	>128	16	32	4	8	8
China-HB12	0.25	0.125	>256	32	1	>128	>128	16	32	4	8	8
China-HB13	0.25	1	256	256	1	≤0.06	32	1	1	0.125	<0.06	16
China-HB14	0.06	0.25	>256	32	1	128	64	32	32	0.125	1	16
China-HB15	0.5	1	>256	>256	1	≤0.06	8	0.5	1	1	8	16
South Korea-1	0.25	0.125	>256	>256	0.5	>128	>128	16	32	1	1	32
South Korea-2	0.125	0.125	>256	>128	0.5	>128	64	8	16	8	1	16
South Korea-3	0.125	0.5	>256	>256	0.5	>128	>128	32	16	32	1	16
China-HK1	0.25	0.5	>256	128	0.5	1	>128	16	64	16	≥128	8
China-HK2	0.125	0.5	>256	>128	0.5	64	128	8	16	16	2	8
China-HN1	0.047	0.25	>256	>256	1	64	>128	4	32	4	8	8
China-HN2	≤0.06	0.25	>128	>128	1	128	>128	64	64	4	8	16
China-HN3	0.125	0.25	>128	>128	1	≤0.06	64	16	32	≤0.06	4	16
China-HN4	≤0.06	0.25	>128	>128	1	≤0.06	>128	8	64	1	1	8
China-HN5	0.125	0.25	>128	>128	1	≤0.06	4	1	1	1	4	8
China-HN6	0.125	0.25	>128	>128	2	>128	128	16	32	4	8	16
China-JX1	≤0.06	0.125	16	0.125	1	64	32	16	16	2	4	8
China-HZ1	0.25	0.125	>256	>128	1	≤0.06	>128	16	32	0.125	1	8
China-HZ2	0.125	0.25	>256	>128	2	2	64	16	64	0.125	1	8
China-HZ3	0.125	0.5	>128	>128	2	1	8	1	2	1	<0.06	32
Singapore-1	0.25	0.25	>128	>128	2	≤0.06	4	0.5	0.5	0.25	1	16
China-HZ4	0.06	0.25	>256	>128	2	32	32	16	32	1	1	16
China-YY1	0.125	0.25	>256	>128	1	1	1	1	0.5	0.25	1	1
China-YZ1	0.25	0.25	>128	>128	2	<0.06	4	2	1	8	2	4
China-HZ5	0.25	0.125	>256	>128	0.5	1	>128	1	1	16	<0.06	16
China-HZ6	0.125	0.125	>128	>128	1	1	>128	16	32	16	1	8
China-HZ7	0.25	0.125	2	4	1	≤0.06	1	0.5	1	0.25	<0.06	1
China-HZ8	0.25	0.25	>128	>128	0.5	2	>128	0.5	64	1	1	16
China-HZ9	0.125	0.125	>128	>128	2	128	>128	16	16	2	1	16
China-HZ10	0.125	0.25	>256	>128	1	2	1	0.5	1	2	<0.06	2
China-HZ11	0.125	0.25	>256	>128	1	2	64	0.5	4	4	<0.06	8
New York-1	0.125	0.25	>128	128	1	1	4	1	1	4	4	0.5
New York-2	≤0.06	0.25	>128	128	1	≤0.06	2	1	1	2	0.5	8
New York-3	0.125	0.25	128	128	1	≤0.06	4	1	1	2	4	1
New York-4	≤0.06	0.5	128	128	2	1	4	0.5	0.5	2	4	8
New York-5	0.125	0.25	128	>128	1	≤0.06	4	1	1	4	2	8
New York-6	0.125	0.25	>128	128	1	≤0.06	>128	0.5	1	2	2	0.25
New York-7	0.25	0.25	64	128	1	≤0.06	4	1	1	2	0.5	32

^a^ Metronidazole (MTZ), vancomycin (VAN), clindamycin (CLI), erythromycin (ERY), fusidic acid (FuA), rifampicin (RIF), levofloxacin (LFX), moxifloxacin (MFX), gatifloxacin (GAT), tetracycline (TET), piperacillin/tazobactam (PIP-TAZ), and ciprofloxacin (CFX).

The antibiotic phenotype-genotype concordance was further examined. A total of 55.6% (25/45) and 2.2% (1/45) fluoroquinolone resistant ST37 isolates harboured the T82I and E53K mutations in *gyrA*, while 13.3% (6/45) and 2.2% (1/45) contained D465N and R447K substitutions in *gyrB*, respectively. All 45 fluoroquinolone-resistant ST37 isolates contained the *cdeA* gene and V426D in *gyrB*. Overall, the fluoroquinolone genotype showed a 93.8% (45/48) concordance with the fluoroquinolone phenotype. Moreover, the mutation (C656T) in 23S rRNA gene existed in all 48 isolates, in which 72.9% (35/48) had the *ermB* gene. Thus, at least one MLS_B_ resistance determinant was detected in all 48 isolates, showing a concordance rate of 97.9% (47/48) between antibiotic phenotype and genotype. However, the remaining one MLS_B_ susceptible isolate also carried the *ermB* gene and the mutation (C656T), suggesting alternative factors might be involved. One or more rifampicin-resistant substitutions (H502N and R505K in *rpoB*) were found in the 20 isolates, of which 19 (95%) were resistant to rifampicin*.* The tetracycline genotyping was a poor predictor of tetracycline phenotype, with only 15.6% (7/45) of the *tetM* positive isolates presenting tetracycline resistance (Figure S3).

### CDI severities, toxin expression, and sporulation

**Figure 4.**
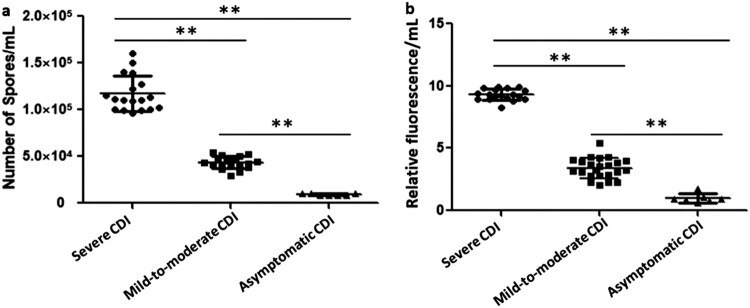
The toxin concentration and sporulation capacity were shown as the means ± standard deviation. Significant differences were marked with **P < 0.05. Comparison of the sporulation determination (a) and toxin B production (b) among the isolates with different clinical symptoms. “●”, “▪” and “▴” represented the average value of three parallel experiments for each strain, respectively.

The clinical information for the 48 patients was shown in Table 2. Based on the ESCMID, SHEA and IDSA guidelines [23,24], 18 (37.5%) patients were diagnosed with severe CDIs, whereas 7 (14.6%) and 23 (47.9%) patients as asymptomatic carriers and mild-to-moderate CDIs, respectively, were classified as milder CDI in Table 2. The results showed that patients over 65 years (64.3%) had higher likelihood to develop severe CDIs as compared to milder CDIs (35.7%) (*χ*^2 ^= 5.70, *P = *0. 017). In addition, severe CDI cases were more frequently found in China than in other countries (Fisher’s exact test, *P = *0.031). CDI severities varied significantly in the four areas (Zhejiang, Hebei, Hunan and Hong Kong) in China (Fisher’s exact test, *P = *0.009), with the highest rate of severe CDI found in Zhejiang. Phylogenetic analysis showed that *C. difficile* isolates from patients with severe CDIs were widely scattered in the Chinese phylogenetic tree, without apparent CDI severity-dependent distributions (Figure 2). Among the 20 isolates clustered into CSL1, 8 (40%) were associated with severe CDI, 9 (45%) with mild-to-moderate CDI, and 3 (15%) were asymptomatic carriers. Similar results were observed for the other two CSLs. The 38.9% (7/18) and 50% (3/6) of strains in CSL2 and CSL3 were respectively associated with severe CDI. Additionally, there were no significant correlations between clinical severities and the regions of isolation (*P* > 0.05). The measurements of toxin expression and sporulation showed that the 18 isolates associated with severe CDI had significantly higher toxin concentrations and stronger sporulation abilities than those with milder CDI (*χ*^2^ = 39.23 and 39.22, respectively; *P *< 0.001) (Figure 4). Our results indicated the clinical CDI severities were positively correlated with *C. difficile* toxin expression and sporulation.

**Table 2.** Clinical information of the 48 patients involved in this study.

Patient characteristics	Clinical information	Severe CDI (*n* = 18)	Milder CDI (*n* = 30)	*χ*^2^	*P*
Demographics					
Age (mean, median [range]) (yr)^a^	58.5, 55.5 (4-86)			5.70	0.017
<65		9 (50%)	24 (82.8%)		
≥65		9 (50%)	5 (17.2%)		
Gender^a^ Male (*n* [%])	27 (57.4%)	11 (61.1%)	16 (55.2%)	0.16	0.689
Isolation place (*n* [%])				Fisher’s exact (between countries)	0.031
China				Fisher’s exact (between provinces in China)	0.009
Zhejiang	14 (29.2%)	10 (55.6%)	4 (13.3%)		
Hebei	15 (31.3%)	3 (16.7%)	12 (40%)		
Hunan	6 (12.5%)	4 (22.2%)	2 (6.7%)		
Hong Kong	2 (4.2%)	0	2 (6.7%)		
South Korea					
Pusan	3 (6.3%)	1 (5.6%)	2 (6.7%)		
Singapore	1 (2.1%)	0	1 (3.3%)		
USA					
New York	7 (14.6%)	0	7 (23.3%)		
Isolation time (*n* [%])				2.71	0.100
2011–2013	22 (45.8%)	11 (61.1%)	11 (36.7%)		
2014–2017	26 (54.2%)	7 (38.9%)	19 (63.3%)		
Ward type (*n* [%])				Fisher’s exact test	0.283
Oncology	10 (20.8%)	6 (33.3%)	4 (13.3%)		
Gastroenterology	5 (10.4%)	1 (5.6%)	4 (13.3%)		
Intensive care unit	7 (14.6%)	5 (27.8%)	2 (6.7%)		
Surgery	4 (8.3%)	2 (11.1%)	2 (6.7%)		
Infectious disease	2 (4.2%)	1 (5.6%)	1 (3.3%)		
Others	12 (25%)	3 (16.7%)	9 (30%)		
Non-ward type (*n* [%])					
Physical exam centre	2 (4.2%)	0	2 (6.7%)		
Kindergarten	5 (10.4%)	0	5 (16.7%)		
Unknown	1 (2.1%)	0	1 (3.3%)		

^a^ The clinical information of one sample from Singapore is absent.

### Identification of gene mutations associated with severe CDI

Comparative analysis of the 48 *C. difficile* genomes identified 71 unique missense mutations (50 SNPs and 21 indels) in 60 genes, from the 18 isolates associated with severe CDI (Tables S5 and S6). These mutations were annotated based on the M68 reference genome, and the gene positions and functions, with forward and reverse open reading frames, were shown in Figure S4a and S4b.

**Figure 5.**
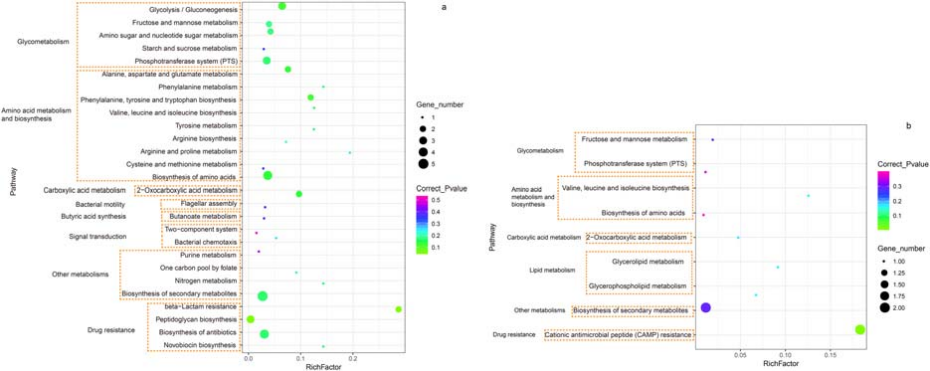
The enriched Kyoto Encyclopedia of Genes and Genomes (KEGG) molecular pathways for genes with mutations among isolates associated with severe CDI. (a) Statistics of enriched pathways involved in the SNPs specific to severe CDI. (b) Statistics of enriched pathways involved in the indels specific to severe CDI. The X- axis was the rich factor. The Y-axis was the enriched pathways with gene functions listed on the left. Coloured dots represented corrected *P* value and the size of black dots indicated the number of genes involved in the metabolic pathway.

KEGG pathway analysis indicated that 21 of the 60 genes were crosswise-associated with a total of 28 metabolic pathways (Figure 5). Although no specific gene mutations were shared within the 18 isolates, mutations among genes involved with the same metabolic pathways have been found in isolates associated with severe CDI. It was possible that these mutations might influence *C. difficile* biological reactions through interfering different genes on the same pathways. KEGG enrichment further showed 10 metabolic pathways, including cationic antimicrobial peptide resistance, glycerolipid metabolism, peptidoglycan biosynthesis, beta-lactam resistance, glycolysis/gluconeogenesis, phenylalanine, tyrosine and tryptophan biosynthesis, biosynthesis of amino acids, valine, leucine and isoleucine biosynthesis, alanine, aspartate and glutamate metabolism and 2-oxocarboxylic acid metabolism, were significantly enriched in the gene mutations among isolates associated with severe CDI (*P *< 0.05) (Figure 5). Five genes with mutations were related to the glycometabolism (GM) including *bglA*, *celF*, *phosphotransferase system (PTS) glucose transporter subunit IIBC*, *PTSmannose transporter subunit IIAB*, and *PTS fructose transporter subunit IIC* in the 3 isolates. Of them, a gene mutation in *bglA* was found in one isolate, two gene mutations in *celF* and *PTS glucose transporter subunit IIBC* occurred in the same isolate, and mutations in the genes of *PTS mannose transporter subunit IIAB* and *PTS fructose transporter subunit IIC* occurred in another same isolate. Similarly, five genes with mutations associated with amino acid metabolism and biosynthesis (AAMB) including *aroK*, *aspartate aminotransferase*, *leuC*, *leuD*, and *aspB* were found in the 5 different isolates (each gene mutation in one isolate). Other gene mutations in the eight enriched metabolic pathways were presented in Table S5 and Table S6.

### Relative mRNA expression levels of the toxin B and its regulatory genes

The CcpA and CodY as transcription factors are associated with toxin synthesis mediated by glucose and BCAAs, respectively [44,45]. Therefore, expressions of the toxin gene (*tcdB*) and the toxin regulatory genes (*tcdR*) were quantified in all 48 isolates. The relative mRNA levels of *tcdB* (8.47 ± 0.55) and *tcdR* (9.87 ± 0.75) were significantly higher in the *C. difficile* isolates from severe CDI patients with mutations than that in those with non-severe CDIs including mild to moderate CDI (*tcdB*: 1.58 ± 0.37; and *tcdR*: 0.41 ± 0.29, *Z* = −0.53, *P *< 0.001) and asymptomatic carriage (*tcdB*: 1.73 ± 0.11; and *tcdR*: 0.48 ± 0.18, *Z* = −3.74, *P *< 0.001) based on real-time RT–PCR. These results indicated that severe clinical CDIs were associated with high *tcdB* mRNA (Figure S5a) expressions*,* and that *tcdR* was positively correlated with *tcdB* at the transcriptional level (Figure S5b). There were no significant differences among the isolates from severe CDI patients in the relative mRNA expression levels of *tcdB* and *tcdR* (*P >* 0.05) (Figures S5a and S5b).

We also analysed the transcription levels of *ccpA* and *codY* in the *C. difficile* isolates of severe CDIs with mutation genes encoding GM (*n* = 3) and AAMB (*n* = 5), isolates with mild to moderate CDI and asymptomatic carriage, and other severe CDI, respectively. The relative mRNA expression level of *ccpA* was significantly lower in the isolates with mutation genes encoding GM causing severe CDI (0.35 ± 0.02) than that in the isolates associated with mild to moderate CDI (1.88 ± 0.50, *P *< 0.001), asymptomatic carriage (1.84 ± 0.11, *P *< 0.001), and other severe CDI (1.77 ± 0.33, *P *< 0.001) (Figure S5c). Similar results were observed for *codY*, with significant differences observed between the severe (0.45 ± 0.16) groups with mutation genes encoding AAMB and others including mild to moderate CDI (2.52 ± 0.38, *Z* = −3.46, *P = *0.0005), asymptomatic carriage (2.67 ± 0.26, *Z=*−3.85, *P =* 0.0044) and other severe CDI without AAMB (2.18 ± 0.25, *Z =* −3.06*, P =* 0.0022), respectively (Figure S5d). The results indicated that the *ccpA* and *codY* were significantly inhibited in isolates with gene mutations associated with GM and AAMB.

## Discussion

The prevalence of clinically relevant cases of infection caused by A^−^B^+^ *C. difficile* has been increasing worldwide in recent years [4,5,11,46]. A^−^B^+^ ST37 (RT017) is one of the most dominant genotypes associated with severe CDIs in China [6,10,47,48]. Global phylogeny and intercontinental transmission of ST37 strains have been described previously [18], whereas, the transmission routes and genomic characteristics of ST37 isolates in China, the ST37 endemic region, remain largely unexplored. To our knowledge, this is the first study to analyse the phylogeny and transmission routes of ST37 isolates in China. The detailed genomic characteristics and phenotypes of ST37 isolates associated with severe CDIs in China were also determined.

*C. difficile* genome presents high levels of gene diversity, remarkable plasticity, and ultralow levels of gene conservation [14,49]. Horizontal gene transfer and large-scale homologous recombination are two essential mechanisms underlying its genomic evolution as our data indicated [49]. Resistance to antibiotics in *C. difficile* is mediated predominantly by Tns as opposed to plasmids in other pathogens [14]. Since the acquisition of Tn*916*-like transposon, a number of other transposons harbouring different antibiotic resistance genes were subsequently obtained by ST37 genomes within approximately 40 years of evolutionary history, presumably due to various antibiotic selection pressures. The repetitive acquisitions of transposable elements have driven the genetic diversity in the genome evolution of the global ST37 strains. Interestingly, our study revealed that transposons (Tn*916*, Tn*6247*, Tn*6248*, and Tn*5251*) carrying the *tetM* gene were found in almost all ST37 isolates (91.7%, 298/325) since 1977, which was obviously higher than that (65.0%) reported previously [16]. The high detection rate of *tetM*, which mediates tetracycline resistance, suggested that this gene has significantly contributed to the emergence and transmission of ST37 strains in the early stage (∼1970-1980s). Tetracycline was initially introduced into clinical treatment around over half a century ago and were mainly replaced by fluoroquinolones in human medicine due to the emergence of resistance [16]. Thus, the wide usage of tetracyclines in clinical treatment in this timeframe might drive the emergence of tetracycline resistant *C. difficile* by acquiring the *tetM* gene under the antibiotic selection pressure. Homologous recombination, another mechanism of the *C. difficile* genome evolution, was also found to significantly drive ST37 genome evolution, which has been reported in other genetic backgrounds of *C. difficile* strains [15,18,25,49]. The *r/m* ratio for *C. difficile* has been reported to range from 0.2 to 1.13, significantly lower than those from other gut pathogens but comparable to that for *Staphylococcus aureus* and *Enterococcus faecalis* [14]. However, the *r/m* ratios for all global *C. difficile* ST37 and Chinese ST37 isolates were approximately 6.66 and 7.49, respectively. These findings indicate that *C. difficile* ST37 has a high genome sequence diversification caused by frequent homologous recombination events, suggesting that there was high rate of homologous recombination in *C. difficile*.

The global transmission routes of *C. difficile* ST1 (RT 027) only involved South Korea in Asia, and this genotype was rarely detected in China [17]. However, ST37 (RT017) has been currently one of the most predominant genotypes in China [10,13]. Our study showed that *C. difficile* ST37 in mainland China formed three independent and stably transmitted sublineages, derived from the same most recent common ancestor (MRCA), diverging in approximately 1985. Furthermore, our data suggested that Chinese ST37 isolates likely originated from European and Japanese strains, which in turn originated in North America. Bayesian evolutionary analysis showed that *C. difficile* ST37 existed before the 1970s, suggesting that it might predate the North American origin as previously described [18]. Even though the previous epidemiological study suggested that ST37 originated in Asia and was then transmitted to other regions of the world [11], the current global genome phylogeny did not seem to support this hypothesis. Further genomic analysis of historic ST37 isolates may help to identify the true geographical origin of the ST37 genotype.

The antibiotic resistance profiles on ST37 have been presented with a high rate (91.7%) of multidrug-resistance. However, no ST37 isolates were found to be heteroresistant or resistant to metronidazole in this study. It has been reported that the heteroresistance to metronidazole in *C. difficile* ST37 was isolated in China before more than ten years ago [50]. We speculated that ST37 isolates might have different metronidazole resistance phenotypes in China. Thus, plan is on the way to collect more *C. difficile* ST37 isolates from other clinical geographic sites in China in order to further investigate metronidazole resistance phenotype of *C. difficile* ST37. As for other antibiotics with high resistance rates, most of antibiotics including MLS_B_, fluoroquinolone, and rifampicin presented good concordance between phenotype and genotype except tetracycline. Tetracycline has been widely used for the treatment of sexually transmitted diseases, control and prevention of infections in animals [16], and as an alternative in the treatment of CDI by presenting lower risk compared to other antibiotics [51]. Even though *tetM*-harboring Tn*916* like transposons have inserted and co-evolved ST37 genomes since 1970s, most of strains totally lost the phenotypic resistance with only 15.6% of the *tetM* positive isolates presenting tetracycline resistance. No other genes associated with tetracycline resistance were found in ST37 genomes. Similarly, a 100% concordance rate between tetracycline genotype and phenotype was reported in ST11 [15], but the reason was that more than one tetracycline resistance genes (*tetO*, *tet-40*, *tet-44*, and etc) except *tetM* existed in genomes and mediated tetracycline resistance under the continuous selection pressure of tetracycline usage in animal husbandry [16]. Unlike the zoonotic ST11 (RT078), ST37 (RT017) was frequently transmitted among humans who selectively consumed tetracycline and its derivatives for over past 50 years due to obvious side effects [52]. Thus, less selection pressure of tetracycline usage might make *tetM* poorly expressed to lead to a low resistance rate of tetracycline in ST37 (RT017) in spite of *tetM* gene existing. However, this hypothesis needs to warrant further confirmation.

*C. difficile* virulence is mainly associated with TcdB, which is regulated by multifarious metabolic pathways [53]. Some regulatory genes mediate toxin gene expression through interactions with *tcdR*, a positive regulator of *tcdB* [54]. In this study, KEGG analysis revealed that all the pathways shown in Figure 5 were involved in pairwise interactions, such as bacterial chemotaxis and flagellar assembly, bacterial chemotaxis and the two-component system, and the two-component system and nitrogen metabolism. Notably, some of them were well known to be involved in the regulation of *C. difficile* toxin gene expression in response to environmental signals. Uptake of rapidly metabolizable carbohydrates, including glucose or other sugars [55], has been described to affect toxin synthesis, which is mediated by the CcpA regulator, a member of the LacI/GalR family of transcription factors [44]. Metabolism and biosynthesis of amino acids, particularly BCAAs, suppresses the toxin synthesis by activating *codY* and triggering the release of CodY [45].

The two regulators, CcpA and CodY, influence the *C. difficile tcdB* expression by binding to the *tcdR* promoter region. Our study indicated that the ST37 isolates associated with severe CDI showed high levels of transcription of *tcdB* and *tcdR*, which is consistent with their clinical severity. In addition, isolates with mutations in the gene for GM and AAMB had low levels of transcription of *ccpA* and *codY*. We hypothesized that these gene mutations might directly or indirectly suppress *ccpA* and *codY* then stimulate *tcdR* expression and promote toxin synthesis, which is consistent with that CcpA mediates repression of toxin production and toxin synthesis enhanced by inactivation of codY [53]. In the near future, further independent experiments on transcript levels of toxin-related genes should be run by testing three different colonies of each ST37 strain in order to confirm the results above. After that, further studies are needed to understand the mechanisms how these gene mutations affect *ccpA* and *codY* expressions*,* and to elucidate the virulence regulatory pathways.

The activation of toxin gene expression in *C. difficile* is responsive to multiple components in the bacterium’s nutritional environment including glucose and branched-chain amino acids (valine, leucine and isoleucine; BCAAs) [53]. Moreover, gene mutations may alter the metabolic capabilities through loss of gene biological functions [56]. Thus, biological functions altered by the gene mutations in *leuC* and *leuD* associated with biosynthesis of BCAAs and *PTS glucose transporter subunit IIBC* gene related with GM were further studied using molecular modelling. We respectively identified P108H substitution in *leuC* gene, A13V substitution in the *PTS glucose transporter subunit IIBC*, and a frameshift in *leuD* in three *C. difficile* strains associated with severe CDI. Their mutant protein sequences were then artificially modelled to assess their influence on the stability of the three-dimensional protein structures. Molecular modelling showed that all the three gene mutations could impact the spatial protein structures, causing instability in protein activity and loss of function (Figure S6).

*C. difficile* virulence is also related to its sporulation ability, which is critical to its survival in the environment or outside the gastrointestinal tract, and plays an important role in driving transmission between different hosts [45]. Enhanced spore formation capability suggests prolonged environmental survival and high risk of transmission. Thereby, it was very important to control and prevent severe CDI caused by *C. difficile* ST37 with strong sporulation abilities. *C. difficile* spore formation is triggered by environmental stimuli, such as nutrient deprivation, population density, and availability of simple sugars [57,58]. Moreover, previous studies indicated that CodY, functioning as a repressor, regulated a panel of genes to suppress the initiation of *C. difficile* spore formation [57,59]. Similarly, CcpA indirectly regulates *C. difficile* sporulation [44]. In this study, ST37 isolates from patients with severe CDI showed high sporulation abilities. Accordingly, the suppression of mRNA expression of *ccpA* and *codY* was found in isolates with mutations in GM and AAMB pathway, which suggested these gene mutations might increase the sporulation abilities through the inhibition of *ccpA* and *codY*. However, the expression inhibition was not observed in other isolates causing severe CDI, suggesting *C. difficile* sporulation in these ST37 isolates were regulated by other mechanisms.

Our study had some limitations as below. Firstly, our results may underestimate the complexity of ST37 evolution and transmission, due to the possibility of unsampled missing links in the transmission chains. Secondly, CDI surveillance is not commonly performed across mainland China, making comprehensive collection of ST37 isolates from different regions difficult. The numbers of several representative strains were not enough, and the number of cases in each group is small in this study, so the results of statistical test might be biased. Thirdly, due to the lack of clinical information from the 277 global genomes, severity-specific gene mutations were not characterized. Lastly, the functions of the SNPs and indels identified in the severe CDI cases were not experimentally examined, and their contributions to the virulence of ST37 warrant further studies.

In conclusion, we described the first comprehensive genomic epidemiological study of epidemic ST37 *C. difficile* in China, and identified the phylogenetic and genomic characteristics, and the transmission routes of Chinese ST37 isolates. Gene mutation and expression profiles associated with severe CDI-related ST37 isolates were explored. Future studies, including genomic surveillance, RNA transcriptome and proteome analyses, genomic editing and animal models, may be utilized to further understand the national and international transmission of ST37 strains, the regulatory pathways of toxin production and sporulation, and functional determination with clinical severity of *C. difficile* ST37 CDIs.

## Supplementary Material

Supplemental Material

## Data Availability

Data of 277 ST37 genomes are available in NCBI database under Bioproject numbers ERP009770 and PRJEB11868. The genomic data of the 48 isolates sequenced in this study were deposited in the NCBI database under study accession number PRJNA591265. The reference genome of *C. difficile* M68 can be found under GenBank FN668375. The Eleven completely assembled and fully annotated *C. difficile* genomes can be found as Table S1.

## References

[1] Martin JS, Monaghan TM, Wilcox MH. *Clostridium difficile infection*: epidemiology, diagnosis and understanding transmission. Nat Rev Gastroenterol Hepatol. 2016 Apr;13(4):206–216. 26956066 10.1038/nrgastro.2016.25

[2] al-Barrak A, Embil J, Dyck B, et al. An outbreak of toxin A negative, toxin B positive *Clostridium difficile*-associated diarrhea in a Canadian tertiary-care hospital. Can Commun Dis Rep. 1999 Apr 1;25(7):65–69. 10344088

[3] Riegler M, Sedivy R, Pothoulakis C, et al. *Clostridium difficile* toxin B is more potent than toxin A in damaging human colonic epithelium in vitro. J Clin Invest. 1995 May;95(5):2004–2011. 7738167 10.1172/JCI117885 PMC295778

[4] Collins DA, Hawkey PM, Riley TV. Epidemiology of *Clostridium difficile* infection in Asia. Antimicrob Resist Infect Control. 2013 Jul 1;2(1):21. 23816346 10.1186/2047-2994-2-21 PMC3718645

[5] Drudy D, Harnedy N, Fanning S, et al. Emergence and control of fluoroquinolone-resistant, toxin A-negative, toxin B-positive *Clostridium difficile*. Infect Control Hosp Epidemiol. 2007 Aug;28(8):932–940. 17620240 10.1086/519181

[6] Du P, Cao B, Wang J, et al. Sequence variation in tcdA and tcdB of *Clostridium difficile*: ST37 with truncated tcdA is a potential epidemic strain in China. J Clin Microbiol. 2014 Sep;52(9):3264–3270. 24958798 10.1128/JCM.03487-13 PMC4313148

[7] Kim J, Kim Y, Pai H. Clinical characteristics and treatment outcomes of *Clostridium difficile* infections by PCR Ribotype 017 and 018 strains. PLoS One. 2016;11(12):e0168849. 28002482 10.1371/journal.pone.0168849 PMC5176314

[8] Goorhuis A, Debast SB, Dutilh JC, et al. Type-specific risk factors and outcome in an outbreak with 2 different *Clostridium difficile* types simultaneously in 1 hospital. Clin Infect Dis. 2011 Nov;53(9):860–869. 21914851 10.1093/cid/cir549

[9] Arvand M, Hauri AM, Zaiss NH, et al. *Clostridium difficile* ribotypes 001, 017, and 027 are associated with lethal C. difficile infection in Hesse, Germany. Euro Surveill. 2009 Nov 12;14(45):19403. 19941785 10.2807/ese.14.45.19403-en

[10] Jin D, Luo Y, Huang C, et al. Molecular epidemiology of *Clostridium difficile* infection in hospitalized patients in eastern China. J Clin Microbiol. 2017 Mar;55(3):801–810. 27974547 10.1128/JCM.01898-16 PMC5328448

[11] Imwattana K, Knight DR, Kullin B, et al. *Clostridium difficile* ribotype 017 - characterization, evolution and epidemiology of the dominant strain in Asia. Emerg Microbes Infect. 2019;8(1):796–807. 31138041 10.1080/22221751.2019.1621670 PMC6542179

[12] Alfa MJ, Kabani A, Lyerly D, et al. Characterization of a toxin A-negative, toxin B-positive strain of Clostridium difficile responsible for a nosocomial outbreak of *Clostridium difficile*-associated diarrhea. J Clin Microbiol. 2000 Jul;38(7):2706–14. 10878068 10.1128/jcm.38.7.2706-2714.2000 PMC87004

[13] Tang C, Cui L, Xu Y, et al. The incidence and drug resistance of *Clostridium difficile* infection in Mainland China: a systematic review and meta-analysis. Sci Rep. 2016 Nov 29;6:37865. 27897206 10.1038/srep37865 PMC5126672

[14] Knight DR, Elliott B, Chang BJ, et al. Diversity and evolution in the genome of *Clostridium difficile*. Clin Microbiol Rev. 2015 Jul;28(3):721–741. 26085550 10.1128/CMR.00127-14 PMC4475645

[15] Knight DR, Kullin B, Androga GO, et al. Evolutionary and genomic insights into *Clostridioides difficile* sequence type 11: a diverse zoonotic and antimicrobial-resistant lineage of global one health importance. MBio. 2019 Apr 16;10(2):e00446-19. 30992351 10.1128/mBio.00446-19 PMC6469969

[16] Dingle KE, Didelot X, Quan TP, et al. A role for tetracycline selection in recent evolution of agriculture-associated *Clostridium difficile* PCR Ribotype 078. mBio. 2019 Mar 12;10(2):e02790-18. 10.1128/mBio.02790-18 PMC6414706 30862754

[17] He M, Miyajima F, Roberts P, et al. Emergence and global spread of epidemic healthcare-associated *Clostridium difficile*. Nat Genet. 2013 Jan;45(1):109–113. 23222960 10.1038/ng.2478 PMC3605770

[18] Cairns MD, Preston MD, Hall CL, et al. Comparative genome analysis and global phylogeny of the toxin variant *Clostridium difficile* PCR Ribotype 017 reveals the evolution of Two independent sublineages. J Clin Microbiol. 2017 Mar;55(3):865–876. 28031436 10.1128/JCM.01296-16 PMC5328454

[19] Wang L, Luo Y, Huang C, et al. Coinfection with 2 *Clostridium difficile* ribotypes in China: A case report. Medicine (Baltimore). 2018 Mar;97(13):e9946. 29595702 10.1097/MD.0000000000009946 PMC5895390

[20] Luo Y, Cheong E, Bian Q, et al. Different molecular characteristics and antimicrobial resistance profiles of *Clostridium difficile* in the Asia-Pacific region. Emerg Microbes Infect. 2019;8(1):1553–1562. 31662120 10.1080/22221751.2019.1682472 PMC6830245

[21] Huang B, Jin D, Zhang J, et al. Real-time cellular analysis coupled with a specimen enrichment accurately detects and quantifies *Clostridium difficile* toxins in stool. J Clin Microbiol. 2014 Apr;52(4):1105–1111. 24452160 10.1128/JCM.02601-13 PMC3993479

[22] Ryder AB, Huang Y, Li H, et al. Assessment of *Clostridium difficile* infections by quantitative detection of tcdB toxin by use of a real-time cell analysis system. J Clin Microbiol. 2010 Nov;48(11):4129–4134. 20720023 10.1128/JCM.01104-10 PMC3020809

[23] Cohen SH, Gerding DN, Johnson S, et al. Clinical practice guidelines for *Clostridium difficile* infection in adults: 2010 update by the society for healthcare epidemiology of America (SHEA) and the infectious diseases society of America (IDSA). Infect Control Hosp Epidemiol. 2010 May;31(5):431–455. 20307191 10.1086/651706

[24] Crobach MJ, Dekkers OM, Wilcox MH, et al. European Society of Clinical Microbiology and Infectious Diseases (ESCMID): data review and recommendations for diagnosing *Clostridium difficile*-infection (CDI). Clin Microbiol Infect. 2009 Dec;15(12):1053–1066. 19929972 10.1111/j.1469-0691.2009.03098.x

[25] Stabler RA, He M, Dawson L, et al. Comparative genome and phenotypic analysis of *Clostridium difficile* 027 strains provides insight into the evolution of a hypervirulent bacterium. Genome Biol. 2009;10(9):R102. 19781061 10.1186/gb-2009-10-9-r102 PMC2768977

[26] Preston MD, Assefa SA, Ocholla H, et al. Plasmoview: a web-based resource to visualise global Plasmodium falciparum genomic variation. J Infect Dis. 2014 Jun 1;209(11):1808–1815. 24338354 10.1093/infdis/jit812 PMC4017360

[27] Bolger AM, Lohse M, Usadel B. Trimmomatic: a flexible trimmer for Illumina sequence data. Bioinformatics. 2014 Aug 1;30(15):2114–2120. 24695404 10.1093/bioinformatics/btu170 PMC4103590

[28] Croucher NJ, Page AJ, Connor TR, et al. Rapid phylogenetic analysis of large samples of recombinant bacterial whole genome sequences using Gubbins. Nucleic Acids Res. 2015 Feb 18;43(3):e15. 25414349 10.1093/nar/gku1196 PMC4330336

[29] Xie C, Mao X, Huang J, et al. KOBAS 2.0: a web server for annotation and identification of enriched pathways and diseases. Nucleic Acids Res. 2011 Jul;39(Web Server issue):W316–W322. http://www.genome.jp/kegg/. 21715386 10.1093/nar/gkr483 PMC3125809

[30] Ai C, Kong L. CGPS: A machine learning-based approach integrating multiple gene set analysis tools for better prioritization of biologically relevant pathways. J Genet Genomics. 2018 Sep 20;45(9):489–504. http://www.genome.jp/kegg/. 30292791 10.1016/j.jgg.2018.08.002

[31] Li H, Handsaker B, Wysoker A, et al. The sequence alignment/map format and SAMtools. Bioinformatics. 2009 Aug 15;25(16):2078–2079. 19505943 10.1093/bioinformatics/btp352 PMC2723002

[32] Stamatakis A. RAxML version 8: a tool for phylogenetic analysis and post-analysis of large phylogenies. Bioinformatics. 2014 May 1;30(9):1312–1313. 24451623 10.1093/bioinformatics/btu033 PMC3998144

[33] Bouckaert R, Heled J, Kühnert D, et al. BEAST 2: a software platform for Bayesian evolutionary analysis. PLoS Comput Biol. 2014 Apr;10(4):e1003537. 24722319 10.1371/journal.pcbi.1003537 PMC3985171

[34] Drummond AJ, Suchard MA, Xie D, et al. Bayesian phylogenetics with BEAUti and the BEAST 1.7. Mol Biol Evol. 2012 Aug;29(8):1969–1973. 22367748 10.1093/molbev/mss075 PMC3408070

[35] Dingle KE, Elliott B, Robinson E, et al. Evolutionary history of the *Clostridium difficile* pathogenicity locus. Genome Biol Evol. 2014 Jan;6(1):36–52. 24336451 10.1093/gbe/evt204 PMC3914685

[36] Roberts AP, Mullany P. A modular master on the move: the Tn916 family of mobile genetic elements. Trends Microbiol. 2009 Jun;17(6):251–258. 19464182 10.1016/j.tim.2009.03.002

[37] Mullany P, Allan E, Roberts AP. Mobile genetic elements in *Clostridium difficile* and their role in genome function. Res Microbiol. 2015 May;166(4):361–367. 25576774 10.1016/j.resmic.2014.12.005 PMC4430133

[38] Clinical and Laboratory Standards Institute (CLSI). Wayne P. Performance standards for antimicrobial susceptibility testing of anaerobic bacteria. 2017. M11-A8 p.

[39] Magiorakos AP, Srinivasan A, Carey RB, et al. Multidrug-resistant, extensively drug-resistant and pandrug-resistant bacteria: an international expert proposal for interim standard definitions for acquired resistance. Clin Microbiol Infect. 2012 Mar;18(3):268–281. 21793988 10.1111/j.1469-0691.2011.03570.x

[40] Qin J, Dai Y, Ma X, et al. Nosocomial transmission of *Clostridium difficile* genotype ST81 in a general teaching Hospital in China traced by whole genome sequencing. Sci Rep. 2017 Aug 29;7(1):9627. 28851988 10.1038/s41598-017-09878-8 PMC5575120

[41] Antunes A, Martin-Verstraete I, Dupuy B. CcpA-mediated repression of *Clostridium difficile* toxin gene expression. Mol Microbiol. 2011 Feb;79(4):882–899. 21299645 10.1111/j.1365-2958.2010.07495.x

[42] Haynes W. Benjamini–Hochberg method. In: Dubitzky W, Wolkenhauer O, Cho K-H, et al. editors. Encyclopedia of systems biology. New York (NY): Springer New York; 2013. p. 78.

[43] Livak KJ, Schmittgen TD. Analysis of relative gene expression data using real-time quantitative PCR and the 2(-Delta Delta C(T)) method. Methods. 2001 Dec;25(4):402–408. 11846609 10.1006/meth.2001.1262

[44] Antunes A, Camiade E, Monot M, et al. Global transcriptional control by glucose and carbon regulator CcpA in *Clostridium difficile*. Nucleic Acids Res. 2012 Nov;40(21):10701–10718. 22989714 10.1093/nar/gks864 PMC3510511

[45] Daou N, Wang Y, Levdikov VM, et al. Impact of CodY protein on metabolism, sporulation and virulence in *Clostridioides difficile* ribotype 027. PLoS One. 2019;14(1):e0206896. 30699117 10.1371/journal.pone.0206896 PMC6353076

[46] Elliott B, Squire MM, Thean S, et al. New types of toxin A-negative, toxin B-positive strains among clinical isolates of *Clostridium difficile* in Australia. J Med Microbiol. 2011 Aug;60(Pt 8):1108–1111. 21393460 10.1099/jmm.0.031062-0

[47] Yan Q, Zhang J, Chen C, et al. Multilocus sequence typing (MLST) analysis of 104 *Clostridium difficile* strains isolated from China. Epidemiol Infect. 2013 Jan;141(1):195–199. 22475233 10.1017/S0950268812000453 PMC9170587

[48] Chen YB, Gu SL, Wei ZQ, et al. Molecular epidemiology of *Clostridium difficile* in a tertiary hospital of China. J Med Microbiol. 2014 Apr;63(Pt 4):562–569. 24344206 10.1099/jmm.0.068668-0

[49] He M, Sebaihia M, Lawley TD, et al. Evolutionary dynamics of *Clostridium difficile* over short and long time scales. Proc Natl Acad Sci U S A. 2010 Apr 20;107(16):7527–7532. 20368420 10.1073/pnas.0914322107 PMC2867753

[50] Huang H, Weintraub A, Fang H, et al. Antimicrobial susceptibility and heteroresistance in Chinese *Clostridium difficile* strains. Anaerobe. 2010 Dec;16(6):633–635. 20849968 10.1016/j.anaerobe.2010.09.002

[51] Tariq R, Cho J, Kapoor S, et al. Low risk of primary *Clostridium difficile* infection With tetracyclines: a systematic review and metaanalysis. Clin Infect Dis. 2018 Feb 1;66(4):514–522. 29401273 10.1093/cid/cix833

[52] Larkin R. Side-effects of tetracycline alone and of tetracycline with nystatin. Lancet. 1959 Jun 13;1(7085):1228–1229. 13666023 10.1016/s0140-6736(59)90900-6

[53] Bouillaut L, Dubois T, Sonenshein AL, et al. Integration of metabolism and virulence in *Clostridium difficile*. Res Microbiol. 2015 May;166(4):375–383. 25445566 10.1016/j.resmic.2014.10.002 PMC4398617

[54] Mani N, Lyras D, Barroso L, et al. Environmental response and autoregulation of *Clostridium difficile* TxeR, a sigma factor for toxin gene expression. J Bacteriol. 2002 Nov;184(21):5971–5978. 12374831 10.1128/JB.184.21.5971-5978.2002 PMC135396

[55] Collins J, Robinson C, Danhof H, et al. Dietary trehalose enhances virulence of epidemic *Clostridium difficile*. Nature. 2018 Jan 18;553(7688):291–294. 29310122 10.1038/nature25178 PMC5984069

[56] Rohmer L, Hocquet D, Miller SI. Are pathogenic bacteria just looking for food? metabolism and microbial pathogenesis. Trends Microbiol. 2011 Jul;19(7):341–348. 21600774 10.1016/j.tim.2011.04.003 PMC3130110

[57] Zhu D, Sorg JA, Sun X. *Clostridioides difficile* biology: sporulation, germination, and corresponding therapies for *C. difficile* infection.. Front Cell Infect Microbiol. 2018;8:29. 29473021 10.3389/fcimb.2018.00029 PMC5809512

[58] Kumar N, Browne HP, Viciani E, et al. Adaptation of host transmission cycle during *Clostridium difficile* speciation. Nat Genet. 2019 Sep;51(9):1315–1320. 31406348 10.1038/s41588-019-0478-8

[59] Nawrocki KL, Edwards AN, Daou N, et al. CodY-Dependent regulation of sporulation in *Clostridium difficile*. J Bacteriol. 2016 Aug 1;198(15):2113–2130. 27246573 10.1128/JB.00220-16 PMC4944225

