# Supplementary material for: Genomic evolution and virulence association of Clostridioides difficile sequence type 37 (ribotype 017) in China
Source: Emerg Microbes Infect. 2021 Jun 14;10(1):1331–45. doi: 10.1080/22221751.2021.1943538 (PMC8253194; doi:10.1080/22221751.2021.1943538)
Supplement: Supplemental Material [file TEMI_A_1943538_SM6219.docx]

**Figure Legend**

**Fig. S1.** The phylogenetic tree from 325 ST37 genomes based on core SNPs were shown within the center. The inner ring was colored according to geographical locations. The second blue ring was colored according to Indel frequencies. The different Indel frequencies (more than 15%, 10-15%, 5-10%, less than 5%) were showed along with the blue gradient change from deep to light. The purple gradient color of the third ring showed the core genome SNPs frequencies.

**Fig. S2**. Summary of gene variations in ST37 genome. Heatmap (centre): density of recombination events detected within the ST37 genomes, against the M68 reference genome, inferred from the SNPs using Gubbins. (a) The evolution of the Chinese ST37 isolates through time was presented in Fig. 1. Five transposons were found to have exact inserting times as labelled, and the remaining transposons were scattered widely in ST37 genomes. (b) The recombination events occurred in the positions of ST37 genome. Thirteen transposons were showed in the middle part.

Fig. S3. The percentage of phenotypic-genotypic concordance. Blue: antibiotic-resistance related SNPs and genes; green: antibiotic resistance phenotype; orange: the antibiotic resistance phenotype-genotype concordance.

**Fig. S4**. The concentric circles represented the followings (from outside to inside): The two outer rings composed of small colorful lines indicated the open reading frames annotated on the forward and reverse strands of the reference M68 genome, respectively. The loci inside represented whole-genome distributions of unique SNPs and indels specific to severe CDI (outer orange ring and inner green ring). G+C content (plotted using a 10-kb window); GC deviation: (G–C)/(G+C) plotted using a 10-kb window; green indicates values >0, Orange <0). The right part indicated the genes with SNPs and indels participated in the metabolic pathways.

**Fig. S5.** Relative mRNA transcriptions of *tcdB* and [toxin expression regulatory gene](app:ds:toxin%20expression%20regulatory%20gene)s. The relative mRNA transcriptions of *tcdB* (a), *tcdR* (b), *ccpA* (c), and *codY* (d) were measured by using the real time quantitative RT-PCR. *C. difficile* isolates were divided into six groups according to clinical CDI severities. GM: glycometabolism, AAMB: amino acid metabolism and biosynthetic pathway, positive control: ATCC 1870 (ST1, ribotyping 027), negative control: ATCC700057(ribotyping 038). The isolates without mutations were separated from the isolates with GM and AAMB mutations in panel c and panel d, respectively. The relative mRNA expression levels of the six groups were shown as the means ± standard deviation. Significant differences were marked with ***P <* 0.05. “●”represented the average value of three parallel experiments for each strain.

**Fig. S6**. **(a)** Model structure of *leuC* gene with a non-synonymous mutation of P108H. The green helix, arrows and lines represent α-helix, β-sheet, and loops. The side chains of amino acids were shown in a stick model. P108 (red in a1) was close to Y237 (magenta); P mutating to H (blue in a2) crashed the α-helix (yellow) and destroyed the structure of the protein, greatly affecting protein folding and function. **(b)** *leuD* was a homologous dimer. A base inserted before the termination codon does not affect its amino acid sequence, however might affect the formation of protein dimerization. **(c)**: The gene mutation from A (red in c1) to V (blue in c2) in PTS glucose transporter subunit IIBC at site 13 will increase the steric hindrance of this site, which may cause the alpha helix crash on the edge the site and influence the protein conformational instability.


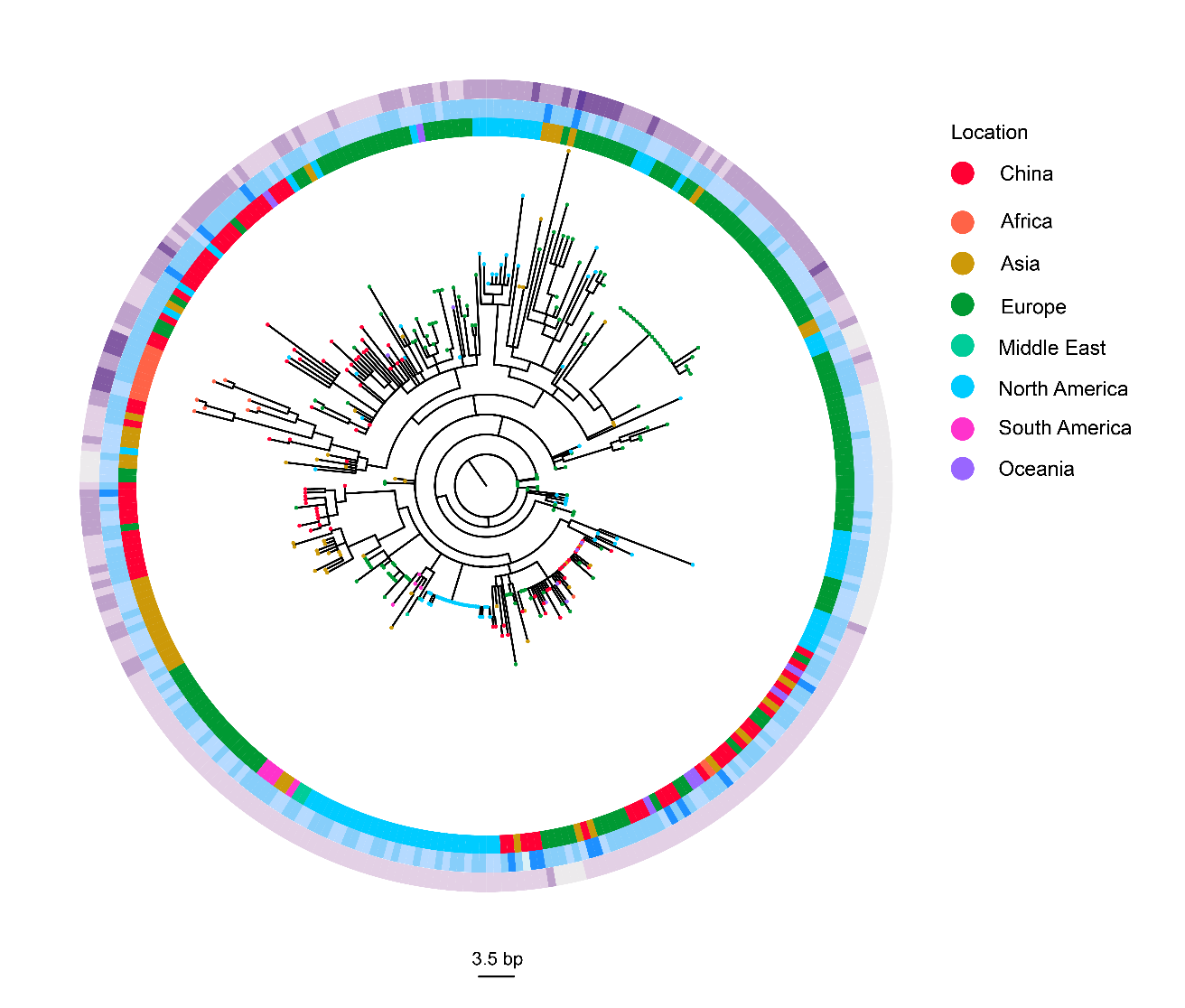


Fig. S1. ML-phylogeny, SNPs, and indels on *C. difficile* ST37 genomes


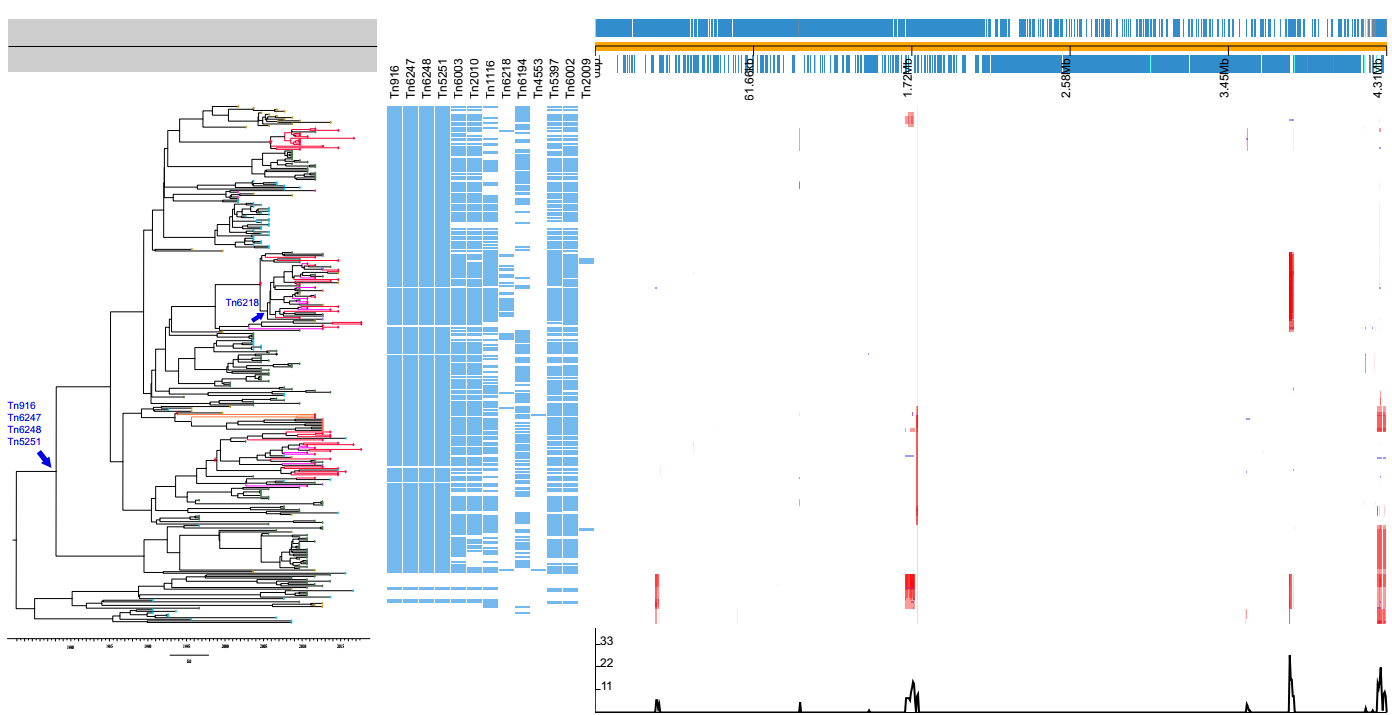


Fig. S2. Summary of gene variations in ST37 genomes

Fig. S3. The percentage of antibiotic resistance phenotypic-genotypic concordance


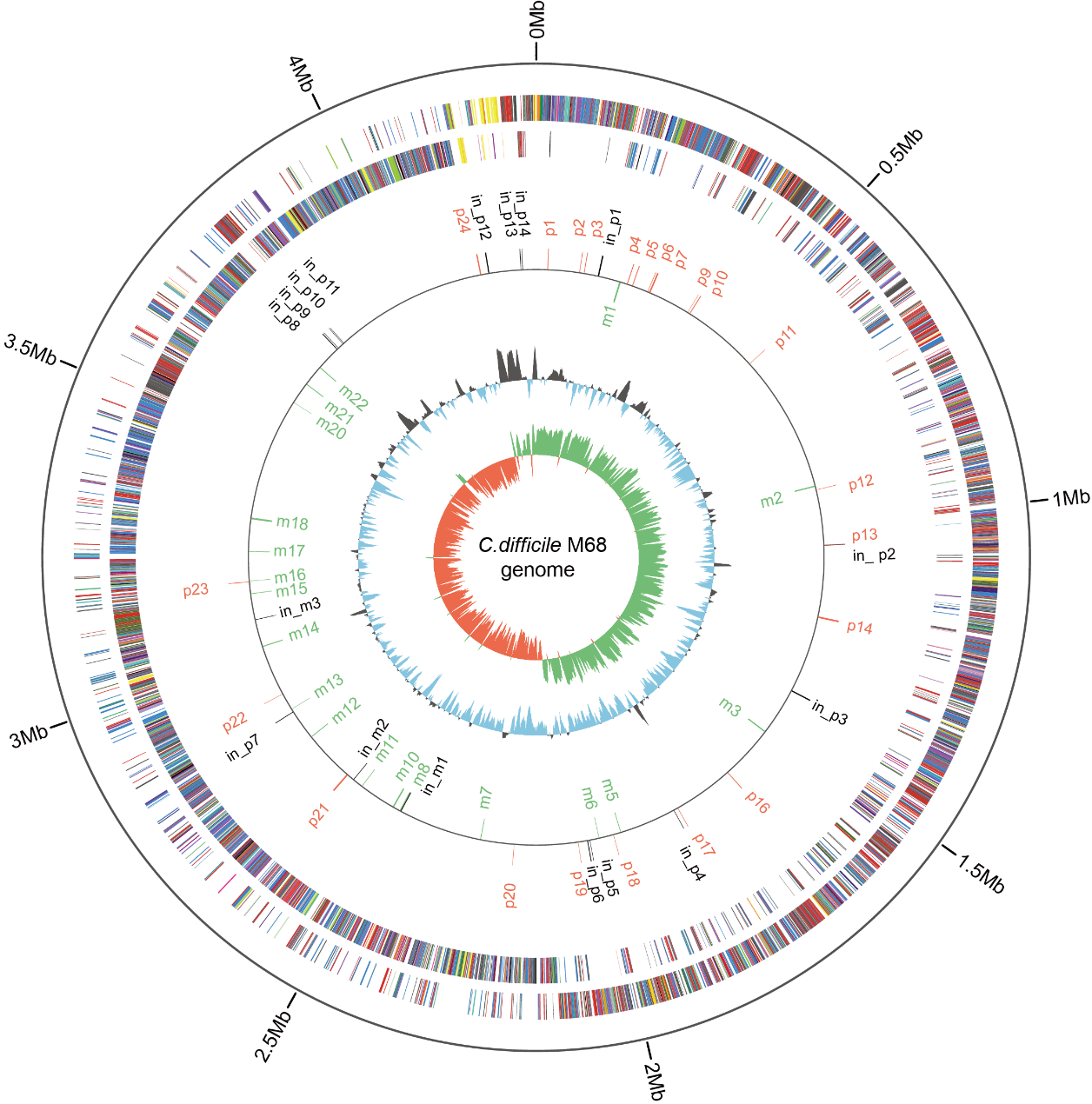


a

b


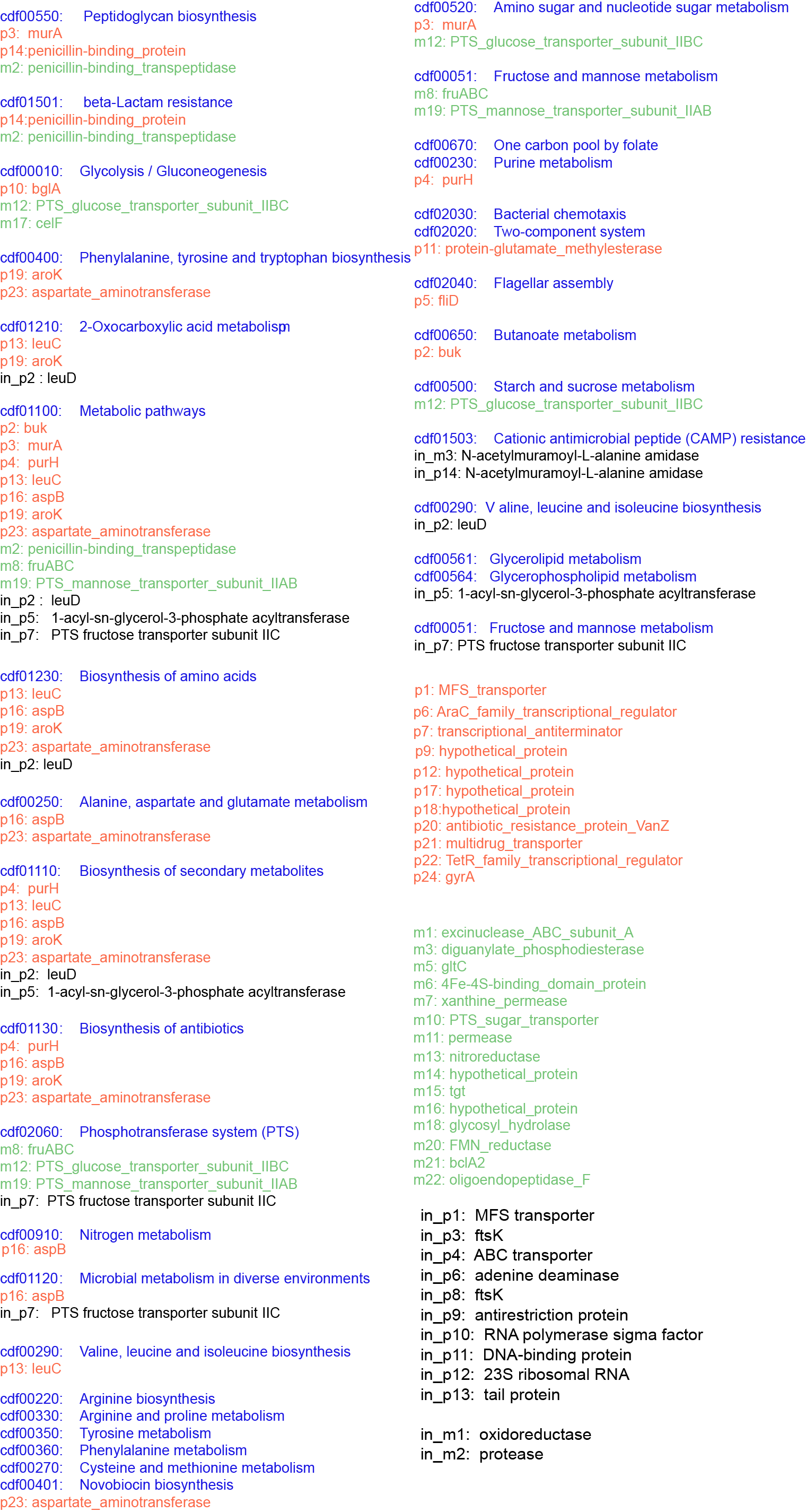


Fig. S4. Location and biological function of severe CDI related gene mutations in the circular *C. difficile* genome.


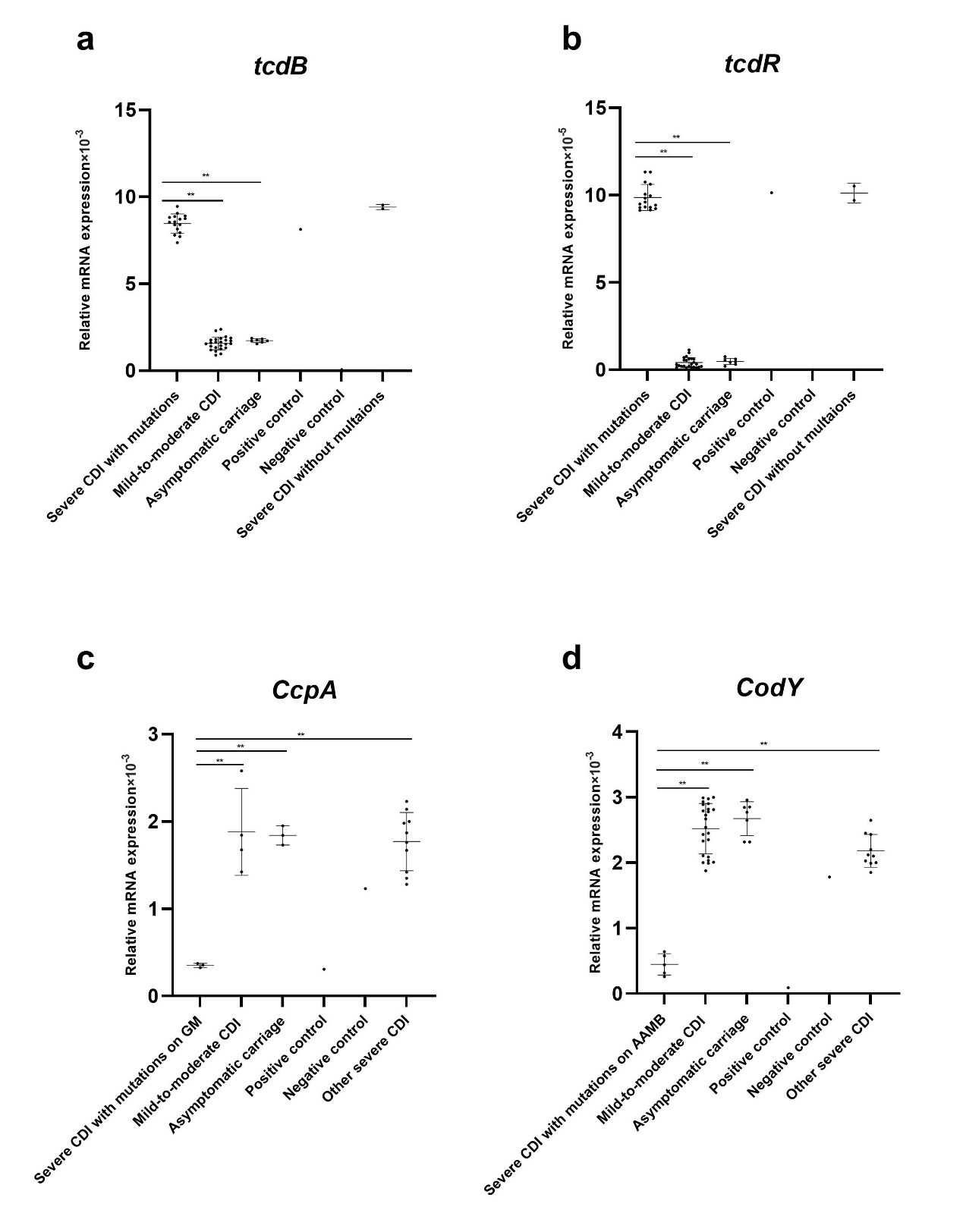


***tcdR***

***tcdB***

***codY***

***ccpA***

Fig. S5. Relative mRNA transcriptions of *tcdB* and toxin expression regulatory genes


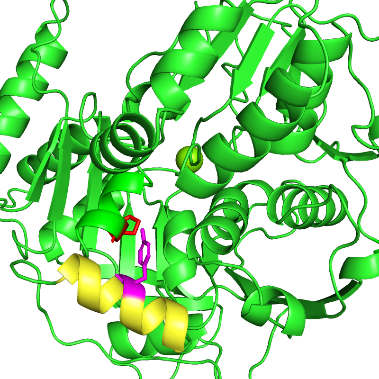

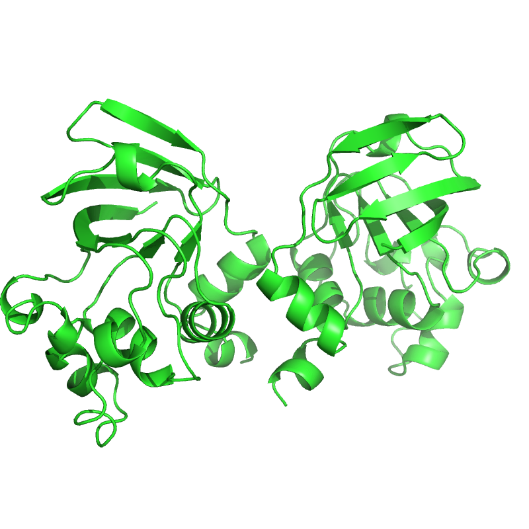

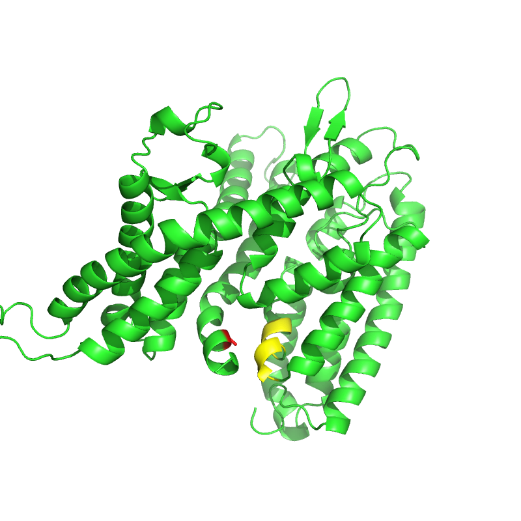

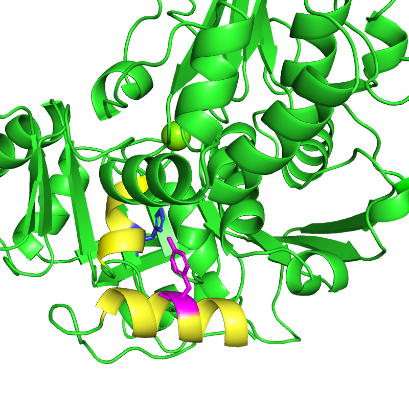

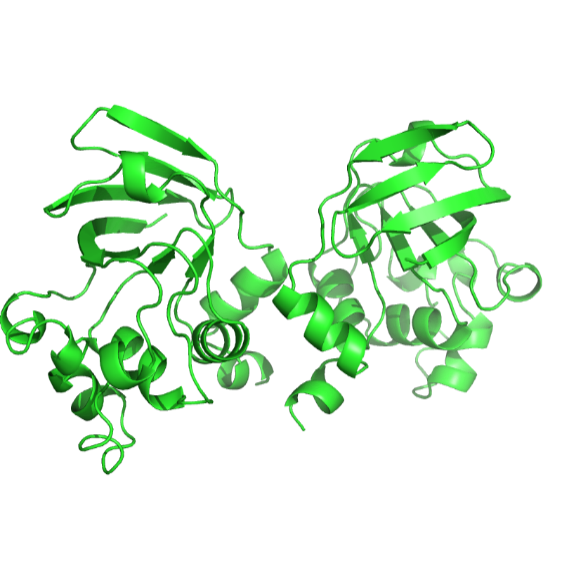

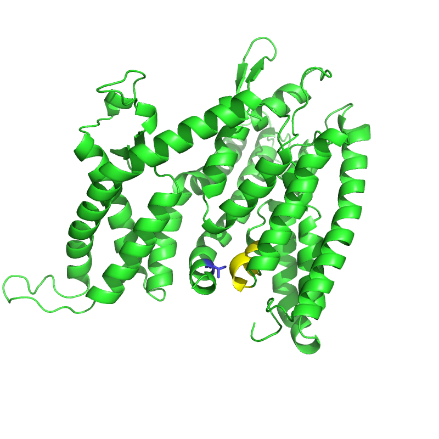


a1 b1 c1

a2 b2 c2

Fig. S6. The model structures of three genes with severe CDI associated mutation

| GenBank accession number | Strain |
| --- | --- |
| FN668375.1 | M68 |
| AM180355.1 | 630 |
| FN538970.1 | CD196 |
| FN665653.1 | M120 |
| FN668941.1 | BI1 |
| CP011968.1 | ATCC 9689 = DSM 1296 |
| CP013196.1 | Z31 |
| CP010888.1 | 08ACD0030 |
| CP019870.1 | BR81 |
| CP020424.1 | FDAARGOS_267 |
| CP022524.1 | DH/NAP11/106/ST-42 |

Table S1. Eleven completely assembled and fully annotated C. difficile genomes

Table S2. PCR primers used in this study.

| Gene target | Primer sequence (5’-3’) | | Amplicon size (bp) | Reference |
| --- | --- | --- | --- | --- |
|  | Forward | Reverse |  |  |
| 16S rRNA | GTGCCTCAGCGTCAGTTACA | GGGAGACTTGAGTGCAGGAG | 119 | [1] |
| *tcdB* | CTGGAGAATGGAAGGTGGTT | TTGATGGTGCTGAAAAGAAGTG | 70 | [1] |
| *tcdR* | GAGGAAGATATTTGTTTTTTCG | AGCCTTATTAACAGCTTGTCTAGATAT | 146 | This study |
| *codY* | ACAAACAAGTGGTGGAAGCAGT | AACTGAACTGTCTTGAGCATC | 141 | This study |
| *ccpA* | GGCTGCCATAGGTGCTATAAATG | TGCTACTGCTCCCATATCGTATAGA | 156 | This study |

**References**

1. Antunes A, Martin-Verstraete I, Dupuy B. CcpA-mediated repression of *Clostridium difficile* toxin gene expression. Mol Microbiol. 2011 Feb;79(4):882-99.

Table S3. Six lineages-defining SNPs

| Lineage | Position^a^ | Amino Acid^a^ | Codon (AA)  M68 Alternative | | Mutation type^b^ | Gene | Gene description | Number of isolates |
| --- | --- | --- | --- | --- | --- | --- | --- | --- |
| Ⅰ | 3821979 | 97 | GAT (D) | GGT (G) | N | *B3/4 domain protein* | B3/4 domain protein | 31 |
| Ⅱ | 3942067 | 31 | AGT (S) | CGT (R) | N | *protein-tyrosine-phosphatase* | protein-tyrosine-phosphatase | 29 |
| Ⅲ | 24253 | 218 | AGA (R) | AAA (K) | N | *beta-glucosidase* | beta-glucosidase | 59 |
| Ⅲ | 932209 | 346 | GCA (A) | TCA (S) | N | *GGDEF-domain containing protein* | diguanylate cyclase | 59 |
| Ⅳ | 1523368 | 491 | GAT (D) | TAT (Y) | N | *diguanylate phosphodiesterase* | diguanylate phosphodiesterase | 16 |
| Ⅳ | 3436874 | 163 | GCA (A) | GTA (V) | N | *bglG* | transcription antiterminator LicT | 16 |
| Ⅴ | 3083548 | 141 | GGA (G) | TGA (U) | N | *haloacid dehalogenase* | haloacid dehalogenase | 55 |
| Ⅴ | 67225 | 813 | GAC (D) | GAT (D) | S | *rpoC* | DNA-directed RNA polymerase subunit beta' | 43 |
| Ⅴ | 4073841 | 177 | GCA (A) | GTA (V) | N | *metal-binding protein* | metal-binding protein | 43 |
| Ⅵ | 49025 | 380 | TAT (Y) | TAC (Y) | S | *gltX* | glutamate--tRNA ligase | 29 |
| Ⅵ | 921488 | 164 | GCA (A) | GAA (E) | N | *permease* | permease | 29 |
| Ⅵ | 1525932 | 104 | ATA (I) | ATG (M) | N | *MerR family transcriptional regulator* | MerR family transcriptional regulator | 29 |
| Ⅵ | 2718614 | 3 | CGA (R) | CGC (R) | S | *hypothetical protein* | hypothetical protein | 29 |
| Ⅵ | 1220509 | 497 | GTA (V) | CTA (L) | N | *penicillin-binding protein* | penicillin-binding protein | 34 |
| Ⅵ | 923154 | 229 | CCA (P) | CTA (L) | N | *CoA transferase* | CoA transferase | 19 |
| Ⅵ | 1234493 | 162 | GGT (G) | AGT (S) | N | *obgE* | GTPase Obg | 19 |
| Ⅵ | 2289973 | 133 | AAA (K) | ATA (I) | N | *xanthine/uracil permease* | xanthine/uracil permease | 19 |
| Ⅵ | 3829751 | 37 | CCT (P) | CAT (H) | N | *multidrug transporter MatE* | multidrug transporter MatE | 19 |

^a^ Positions refer to those in the M68 genome.

^b^ N: nonsynonymous; S: synonymous.

Table S4. Four Chinese sublineages-defining SNPs

| CSL^a^ | Position^a^ | Amino acid^b^ | Codon (AA)  M68 Alternative | | Mutation type^c^ | Gene | Gene description | Number of isolates |
| --- | --- | --- | --- | --- | --- | --- | --- | --- |
| CSL1 | 24253 | 218 | AGA (R) | AAA (K) | N | *beta-glucosidase* | beta-glucosidase | 22 |
| CSL1 | 932209 | 346 | GCA (A) | TCA (S) | N | *GGDEF-domain containing protein* | diguanylate cyclase | 22 |
| CSL1 | 1221197 | 726 | GCA (A) | GTA (V) | N | *penicillin-binding protein* | penicillin-binding protein | 20 |
| CSL2 | 3083548 | 141 | GGA (G) | TGA (U) | N | *haloacid dehalogenase* | haloacid dehalogenase | 55 |
| CSL2 | 67225 | 813 | GAC (D) | GAT (D) | S | *rpoC* | DNA-directed RNA polymerase subunit beta | 43 |
| CSL2 | 4073841 | 177 | GCA (A) | GTA (V) | N | *metal-binding protein* | metal-binding protein | 43 |
| CSL3 | 1220509 | 497 | GTA (V) | CTA (L) | N | *penicillin-binding protein* | penicillin-binding protein | 34 |
| CSL3 | 1221257 | 746 | TCA (S) | TTA (L) | N | *penicillin-binding protein* | penicillin-binding protein | 13 |
| CSL3 | 1221353 | 778 | GCA (A) | GTA (V) | N | *penicillin-binding protein* | penicillin-binding protein | 17 |
| CSL3 | 1384870 | 473 | ATG (M) | GTG (V) | N | *acd* | mannosyl-glycoprotein endo-beta-N-acetylglucosamidase | 14 |
| CSL3 | 1630948 | 691 | GCT (A) | GTT (V) | N | *feoB* | ferrous iron transporter B | 14 |
| CSL3 | 3238652 | 67 | GCT (A) | ACT (T) | N | *MATE family efflux transporter* | MATE family efflux transporter | 14 |
| CSL3 | 3034622 | 86 | ACT (T) | CCT (P) | N | *DNA-binding response regulator* | DNA-binding response regulator | 11 |
| CSL4 | 1221181 | 721 | TGT (C) | CGT (R) | N | *penicillin-binding protein* | penicillin-binding protein | 13 |

^a^ Chinese sublineages

^b^ Positions refer to those in the M68 genome.

^c^ N: nonsynonymous; S: synonymous.

Table S5. Non-synonymous mutations of severe CDI-specific SNPs

| Position^a^ | Amino Acid^a^ | Codon (AA) | | Mutation type^b^ | Gene | Gene description | Number of isolates |
| --- | --- | --- | --- | --- | --- | --- | --- |
|  |  | M68 Alternative | |  |  |  |  |
| 905352 | 569 | ATG (M) | GTG (V) | N | *penicillin-binding transpeptidase* | penicillin-binding transpeptidase | 2 |
| 1802103 | 144 | ATA (I) | ACA (T) | N | *hypothetical protein* | hypothetical protein | 2 |
| 3725133 | 426 | AAT (N) | AAG (K) | N | *oligoendopeptidase F* | oligoendopeptidase F | 2 |
| 27447 | 306 | GGA (G) | TGA (U) | N | *MFS transporter* | MFS transporter | 1 |
| 103404 | 197 | AAT (N) | ATT (I) | N | *buk* | butyrate kinase | 1 |
| 114960 | 368 | GCA (A) | TCA (S) | N | *murA* | UDP-N-acetylglucosamine 1-carboxyvinyltransferase | 1 |
| 202265 | 664 | TCT (S) | TTT (F) | N | *excinuclease ABC subunit A* | excinuclease ABC subunit A | 1 |
| 219679 | 36 | TTT (F) | GTT (V) | N | *purH* | bifunctional phosphoribosylaminoimidazolecarboxamide formyltransferase/inosine monophosphate cyclohydrolase | 1 |
| 233929 | 439 | GCT (A) | ACT (T) | N | *fliD* | flagellar cap protein FliD | 1 |
| 272209 | 86 | GAG (E) | AAG (K) | N | *AraC family transcriptional regulator* | AraC family transcriptional regulator | 1 |
| 276700 | 183 | GCA (A) | GAA (E) | N | *transcriptional antiterminator* | transcriptional antiterminator | 1 |
| 278430 | 760 | GAG (E) | TAG (U) | N | *transcriptional antiterminator* | transcriptional antiterminator | 1 |
| 379653 | 141 | TCT (S) | TAT (Y) | N | *hypothetical protein* | hypothetical protein | 1 |
| 385833 | 427 | CTT (L) | CCT (P) | N | *bglA* | 6-phospho-beta-glucosidase | 1 |
| 571748 | 64 | GCT (A) | ACT (T) | N | *cheB* | protein-glutamate methylesterase | 1 |
| 905492 | 522 | AAT (N) | AGT (S) | N | *penicillin-binding transpeptidase* | penicillin-binding transpeptidase | 1 |
| 915113 | 174 | TAC (Y) | CAC (H) | N | *hypothetical protein* | hypothetical protein | 1 |
| 916912 | 101 | GTA (V) | ATA (I) | N | *anion permease* | anion permease | 1 |
| 1047464 | 108 | CCT (P) | CAT (H) | N | *leuC* | 3-isopropylmalate dehydratase large subunit | 1 |
| 1221182 | 721 | TGT (C) | TCT (S) | N | *penicillin-binding protein* | penicillin-binding protein | 1 |
| 1221389 | 790 | TTT (E) | TCT (F) | N | *penicillin-binding protein* | penicillin-binding protein | 1 |
| 1524351 | 163 | GGG (G) | GTG (V) | N | *diguanylate phosphodiesterase* | diguanylate phosphodiesterase | 1 |
| 1524507 | 111 | GGT (G) | GTT (V) | N | *diguanylate phosphodiesterase* | diguanylate phosphodiesterase | 1 |
| 1656415 | 463 | AAT (N) | ACT (T) | N | *aspB* | oxidoreductase | 1 |
| 1924831 | 44 | GAT (D) | AAT (N) | N | *hypothetical protein* | hypothetical protein | 1 |
| 1949557 | 220 | AGG (R) | ATG (M) | N | *gltC* | sodium/glutamate symporter family protein | 1 |
| 1967934 | 11 | GAT (D) | GGT (G) | N | *hypothetical protein* | hypothetical protein | 1 |
| 2003122 | 174 | GGT (G) | GTT (V) | N | *4Fe-4S-binding domain protein* | 4Fe-4S-binding domain protein | 1 |
| 2054215 | 158 | GCT (A) | GAT (D) | N | *aroK* | shikimate kinase | 1 |
| 2207017 | 136 | GCT (A) | GTT (V) | N | *antibiotic resistance protein VanZ* | antibiotic resistance protein VanZ | 1 |
| 2261408 | 129 | ACA (T) | GCA (A) | N | *hydroxylase accessory protein YqeC* | hydroxylase accessory protein YqeC | 1 |
| 2287681 | 348 | ACT (T) | ATT (I) | N | *xanthine permease* | xanthine permease | 1 |
| 2337984 | 195 | GAG (E) | TAG (U) | N | *hypothetical protein* | hypothetical protein | 1 |
| 2462100 | 8 | GCA (A) | ACA (T) | N | *hypothetical protein* | hypothetical protein | 1 |
| 2511092 | 425 | GAA (E) | TAA (U) | N | *PTS sugar transporter* | PTS sugar transporter | 1 |
| 2600596 | 120 | GTT (V) | CTT (L) | N | *permease* | permease | 1 |
| 2645112 | 678 | GAA (E) | AAA (K) | N | *multidrug transporter* | multidrug transporter | 1 |
| 2769979 | 13 | GCA (A) | GTA (V) | N | *PTS glucose transporter subunit IIBC* | PTS glucose transporter subunit IIBC | 1 |
| 2850484 | 33 | GCA (A) | TCA (S) | N | *nitroreductase* | nitroreductase | 1 |
| 2892591 | 145 | AGC (S) | ATC (I) | N | *TetR family transcriptional regulator* | TetR family transcriptional regulator | 1 |
| 3015783 | 104 | GCA (A) | GTA (V) | N | *hypothetical protein* | hypothetical protein | 1 |
| 3125623 | 127 | CTT (L) | TTT (F) | N | *slpA* | ATP-binding protein | 1 |
| 3144587 | 114 | GTA (V) | TTA (L) | N | *tgt* | tRNA-guanine (34) transglycosylase | 1 |
| 3172299 | 51 | CCA (P) | TCA (S) | N | *aspartate aminotransferase* | aspartate aminotransferase | 1 |
| 3174650 | 205 | AAA (K) | ACA (T) | N | *hypothetical protein* | hypothetical protein | 1 |
| 3245243 | 255 | CAT (H) | TAT (Y) | N | *celF* | 6-phospho-beta-glucosidase | 1 |
| 3323813 | 442 | CCA (P) | CTA (L) | N | *glycosyl hydrolase* | glycosyl hydrolase | 1 |
| 3325468 | 302 | GCT (A) | GTT (V) | N | *PTS mannose transporter subunit IIAB* | PTS mannose transporter subunit IIAB | 1 |
| 3674525 | 215 | GGA (G) | GAA (E) | N | *bclA2* | collagen-like protein | 1 |
| 4174562 | 53 | GAG (E) | AAG (K) | N | *gyrA* | DNA gyrase subunit A | 1 |

^a^ Positions refer to those in the M68 genome.

^b^ N: nonsynonymous; S: synonymous.

Table S6. Severe CDI-specific indels

| Position^a^ | Codon (AA) | | Indel type and bp number^b^ | Gene | Gene description | Number of isolates |
| --- | --- | --- | --- | --- | --- | --- |
|  | M68 Alternative | |  |  |  |  |
| 148944 | AGG | AG | D1 | *MFS transporter* | MFS transporter | 1 |
| 1048917 | AGGGG | AGGGGG | I1 | *leuD* | 3-isopropylmalate dehydratase small subunit | 1 |
| 1406845 | AATGAAGATGAAGATGAAGATGAA | AATGAAGATGAAGATGAA | D6 | *fstK* | cell division protein FtsK | 1 |
| 1812728 | CATATATATAT | CATATATAT | D2 | *ABC transporter* | ABC transporter | 1 |
| 2026094 | AGGGG | AGGGGG | I1 | *1-acyl-sn-glycerol-3-phosphate acyltransferase* | 1-acyl-sn-glycerol-3-phosphate acyltransferase | 1 |
| 2031550 | TGG | TG | D1 | *ade* | adenine deaminase | 1 |
| 2489621 | ATT | AT | D1 | *oxidoreductase* | oxidoreductase | 2 |
| 2625806 | ATTCATGTTTCAAAATCCAAGTTAGTTCAT | ATTCAT | D24 | *protease* | protease | 1 |
| 2843295 | TGCAATATTTGGAGCTTTAGTAATAGCAATATT | TGCAATATT | D24 | *PTS fructose transporter subunit IIC* | PTS fructose transporter subunit IIC | 1 |
| 3081750 | GTTTTTTTTT | GTTTTTTTTTT | I1 | *N-acetylmuramoyl-L-alanine amidase* | N-acetylmuramoyl-L-alanine amidase | 1 |
| 3785063 | A | AG | I1 | cell division protein *FtsK* | cell division protein FtsK | 1 |
| 3788982 | G | GAT | I2 | antirestriction protein | antirestriction protein | 1 |
| 3804089 | AAC | ATTGATGAAACCATCTGAC | I16 | RNA polymerase sigma factor | RNA polymerase sigma factor | 2 |
| 3804968 | ATTG | AAAAACGACACACAGTTG | I14 | DNA-binding protein | DNA-binding protein | 2 |
| 4196266 | G | GC | I1 | 23S ribosomal RNA | 23S ribosomal RNA | 2 |
| 4196271 | CTT | CT | D1 | 23S ribosomal RNA | 23S ribosomal RNA | 1 |
| 4243607 | G | GT | I1 | 16S ribosomal RNA | 16S ribosomal RNA | 1 |
| 4272456 | TAAA | TAGAAA | I2 | tail protein | tail protein | 1 |
| 4272457 | AAAG | AGGAAG | I2 | tail protein | tail protein | 1 |
| 4272458 | AAG | AAGAG | I2 | tail protein | tail protein | 1 |
| 4276586 | A | AG | I1 | N-acetylmuramoyl-L-alanine amidase | N-acetylmuramoyl-L-alanine amidase | 1 |

^a^ Positions refer to those in the M68 genome;

^b^ D: deletion, I: inserting.
